# Mitochondrial dysfunction in asthma: damage signals sustain airway inflammation and remodeling

**DOI:** 10.3389/fimmu.2026.1883589

**Published:** 2026-10-26

**Authors:** Yuqian Zhang, Lujia Liu, Mengdi Shi, Yiqiao Li, Suning Mu, Yan Wang, Youpeng Wang

**Affiliations:** 1 Heilongjiang University of Chinese Medicine, Harbin, Heilongjiang, China; 2 The Second Affiliated Hospital of Heilongjiang University of Chinese Medicine, Harbin, Heilongjiang, China

**Keywords:** airway inflammation, airway remodeling, asthma, innate immune sensing, mitochondria-derived damage signals, mitochondrial dysfunction, mitochondrial oxidative stress, mitochondrial quality control

## Abstract

Asthma is a heterogeneous disease characterized by chronic airway inflammation and airway remodeling. Growing evidence suggests that mitochondrial dysfunction is not only a consequence of inflammatory injury but may also contribute to disease persistence and progression. In asthma, mitochondrial redox balance, energy metabolism, Ca^2+^ homeostasis, dynamics, biogenesis, and quality control can all become dysregulated. These abnormalities are interconnected and can alter mitochondrial energy supply, structural integrity, and quality maintenance. Mitochondrial damage can increase mitochondrial reactive oxygen species, promote the mislocalization of mitochondrial nucleic acids, alter membrane lipid oxidation and remodeling, and induce the release of mitochondrial proteins and other components. These changes generate diverse mitochondria-derived damage signals that can regulate inflammation-related gene transcription, nucleic acid sensing, and inflammasome activation. More broadly, mitochondrial abnormalities also alter immune cell function, airway epithelial repair, smooth muscle cell accumulation, and extracellular matrix production. Together, these effects may contribute to persistent airway inflammation and airway remodeling. This review summarizes the major forms of mitochondrial dysfunction in asthma. It also examines how these abnormalities generate mitochondria-derived damage signals, engage inflammatory sensing, and alter immune and airway structural cell functions.

## 1 Introduction

Asthma is a heterogeneous disease typically characterized by chronic airway inflammation. Its clinical manifestations include recurrent and variable wheezing, shortness of breath, chest tightness, and cough, together with variable expiratory airflow limitation. Persistent airflow limitation may develop in some patients as the disease progresses (1). The Global Burden of Disease study estimated that approximately 260 million people worldwide had asthma in 2021 (2). Inhaled corticosteroids, bronchodilators, and targeted biologic therapies have improved disease control in some patients. However, some patients continue to experience poor disease control and recurrent exacerbations, often accompanied by persistent airway inflammation and airway remodeling (3–5). Based on predominant inflammatory features, asthma is broadly categorized into type 2-high and type 2-low inflammatory endotypes. These endotypes involve distinct upstream drivers, effector cells, and cytokine pathways (6, 7). Although their upstream drivers and effector pathways differ, disrupted organelle homeostasis and immunometabolic abnormalities may still contribute to the persistence of chronic airway inflammation.

Mitochondria are the principal sites of cellular aerobic energy metabolism. They also participate in redox regulation, ion homeostasis, cellular stress responses, immune signaling, and cell fate determination (8). Mitochondrial architecture, dynamics, and contacts with other organelles further influence respiratory efficiency, ion transport, and mitochondrial homeostasis (9). Mitochondrial dysfunction can increase mitochondrial reactive oxygen species (mtROS) and promote the mislocalization or release of mitochondria-derived damage signals such as mitochondrial DNA (mtDNA). These signals can then engage innate immune sensing and downstream signaling pathways (10, 11). Mitochondrial metabolism also regulates immune-cell activation, differentiation, and effector function. These effects can influence the type and magnitude of airway inflammation in asthma (12, 13).

Existing studies have often examined mitochondrial dysfunction, mitochondria-derived damage signals, or individual immune-sensing pathways separately. The continuum from damage-signal generation and release to immune sensing and cellular effects has received less integrated attention. This review therefore summarizes the major forms of mitochondrial dysfunction in asthma, examines how mitochondria-derived damage signals are generated, released, and sensed, and discusses how mitochondrial abnormalities, including damage-signal pathways, alter immune and airway structural cell functions and contribute to persistent airway inflammation and remodeling.

## 2 Mitochondrial dysfunction in asthma

Normal mitochondrial function depends on the coordination of redox balance, bioenergetics, Ca^2+^ homeostasis, mitochondrial dynamics, biogenesis, and mitophagy. In asthma, these processes can all become dysregulated, manifesting as increased oxidative stress, altered bioenergetics and substrate utilization, disrupted Ca^2+^ homeostasis, and abnormalities in mitochondrial dynamics, biogenesis, and mitophagy. These changes interact with one another and collectively affect mitochondrial energy supply, structural maintenance, and damage clearance.

### 2.1 Mitochondrial oxidative stress

The mitochondrial respiratory chain is located in the inner mitochondrial membrane. Electron transfer through the respiratory chain establishes a proton gradient that drives ATP synthesis (14). During electron transfer and substrate oxidation, multiple mitochondrial redox sites can generate superoxide and H_2_O_2_, and the relative contribution of these sites varies with substrate utilization and respiratory state (15, 16). These reactive oxygen species (ROS) are regulated by mitochondrial antioxidant systems. In the mitochondrial matrix, superoxide dismutase 2 (SOD2) converts superoxide to H_2_O_2_, which is subsequently reduced by glutathione peroxidases and peroxiredoxins. Glutathione peroxidases primarily depend on reduced glutathione (GSH), whereas mitochondrial peroxiredoxins rely mainly on the thioredoxin 2 system for their catalytic cycle (17). At physiological levels, mtROS can participate in redox signaling through the reversible oxidation of protein thiol groups (18). When mtROS production exceeds antioxidant capacity, oxidative modifications can accumulate, and mitochondrial proteins, membrane lipids, nucleic acids, and other components can undergo oxidative damage (19).

Increased mtROS in asthma can arise from altered respiratory-chain function and impaired control of mtROS generation. The respiratory chain is both a major site of ROS generation and a target of oxidative damage. Oxidative stress induced by ragweed pollen extract causes oxidative modification of the complex I core proteins NADH dehydrogenase (ubiquinone) Fe-S protein 1 (NDUFS1) and 2 (NDUFS2) and the complex III core proteins ubiquinol-cytochrome c reductase core protein I (UQCRC1) and II (UQCRC2). Further depletion of individual respiratory-chain subunits showed that reduced UQCRC2 increased ROS production, whereas reduced UQCRC1 did not produce the same effect (20). These findings indicate that damage to different respiratory-chain subunits can have distinct effects on ROS generation. Uncoupling protein 2 (UCP2), located in the inner mitochondrial membrane, also contributes to limiting mtROS generation. In primary bronchial epithelial cells exposed to Aspergillus protease, transforming growth factor beta (TGF-β)/SMAD4 signaling downregulated UCP2, and reduced UCP2 further increased mtROS levels (21). Thus, oxidative damage to respiratory-chain proteins and UCP2 downregulation can both alter mtROS generation, although the functional consequences of respiratory-chain injury vary among individual proteins.

Mitochondrial respiration and redox regulation are also closely linked to the nitric oxide synthase (NOS)–nitric oxide (NO) pathway and arginine metabolism. Freshly isolated airway epithelial cells from patients with obesity-associated asthma show increased basal and maximal mitochondrial respiration. These changes are accompanied by enhanced arginase-mediated arginine metabolism, NOS uncoupling, reduced NO bioavailability, and increased oxidative stress. Functional interventions further show that NO signaling contributes to the regulation of mitochondrial respiration and redox status (22). Asymmetric dimethylarginine (ADMA) also affects this pathway. Interleukin-4 (IL-4) exposure reduces dimethylarginine dimethylaminohydrolase 2, which metabolizes ADMA, and increases intracellular ADMA accumulation. In IL-4-pretreated cells, subsequent ADMA exposure further increases mtROS and peroxynitrite and aggravates mitochondrial injury (23). Thus, altered arginine utilization, NOS coupling, and ADMA metabolism can disrupt NOS–NO regulation and further affect mitochondrial respiration and redox homeostasis.

The extent of mtROS accumulation also depends on mitochondrial antioxidant capacity. Paraoxonase 2 (PON2) is partially localized to mitochondria and contributes to antioxidant protection in airway epithelial cells. PON2 protein levels are reduced in the airway epithelium of patients with obesity-associated asthma (24). PON2 depletion increases cellular susceptibility to oxidative stress and causes greater loss of mitochondrial membrane potential and mitochondrial function after H_2_O_2_ exposure (25). Alterations in the superoxide dismutase (SOD) system provide further evidence of impaired superoxide clearance. Mitochondrial SOD2 activity is reduced in neutrophils from patients with asthma, whereas predominantly cytosolic copper/zinc superoxide dismutase 1 (SOD1) activity does not show the same change (26). Reduced total SOD activity has also been observed in other lung-derived cells from patients with asthma. That study did not distinguish between SOD1 and SOD2 (27). An SOD mimetic attenuates antigen-induced respiratory abnormalities, airway inflammation, and oxidative damage in sensitized guinea pigs (28), supporting a protective effect of enhanced superoxide clearance. SOD2 in asthmatic airways can also undergo oxidative and nitrative modifications (29), indicating that the antioxidant enzyme responsible for superoxide clearance can itself be affected by oxidative stress. These findings support a contribution of impaired SOD-dependent antioxidant defense to oxidative stress in asthma.

After SOD2 converts superoxide to H_2_O_2_, subsequent detoxification of H_2_O_2_ and oxidized membrane lipids depends in part on reducing systems such as GSH. In the airway lining fluid of children with severe asthma, the GSH redox state is shifted toward oxidation and is accompanied by increased H_2_O_2_ and lipid oxidation products (30). GSH redox imbalance has also been observed in airway macrophages (31). Increased membrane lipid oxidation in airway epithelial cells further increases the demand for GSH. The interaction of 15-lipoxygenase-1 (15LO1) with phosphatidylethanolamine-binding protein 1 (PEBP1) promotes the formation of phosphatidylethanolamine (PE) hydroperoxides. Glutathione peroxidase 4 (GPX4) consumes GSH while reducing these oxidized phospholipids. Following IL-13 exposure, increased 15LO1 is accompanied by increased GPX4 and solute carrier family 7 member 11 (SLC7A11), which supports GSH synthesis through cystine uptake. However, intracellular GSH levels and the GSH/oxidized glutathione (GSSG) ratio remain reduced. Reducing 15LO1 expression restores GSH status and is accompanied by decreased GPX4 expression (32). These findings indicate that 15LO1-associated lipid oxidation increases GSH consumption. The increases in GPX4 and SLC7A11 are consistent with greater demand for oxidized phospholipid reduction and GSH replenishment, while the overall GSH redox state remains shifted toward oxidation.

Lipid oxidation and GSH consumption also involve mitochondrial components. 15LO1, PEBP1, and GPX4 are detectable in mitochondrial fractions of airway epithelial cells, and oxidized PE species increase in these fractions after IL-13 exposure. Freshly isolated airway epithelial cells from patients with asthma also show increased 15-hydroperoxyeicosatetraenoic acid–phosphatidylethanolamine (15-HpETE-PE), a 15LO1-derived hydroperoxy-PE species. The ratio of 15-HpETE-PE to its corresponding hydroxy product, 15-hydroxyeicosatetraenoic acid–phosphatidylethanolamine (15-HETE-PE), is further increased in patients with severe asthma (33). These findings indicate that 15LO1–PEBP1-associated oxidized PE formation and GPX4-dependent reduction can occur in mitochondrial fractions, while the balance of oxidized PE metabolism in asthmatic airway epithelial cells shifts further toward oxidation. Mitochondrial GSH levels also depend on GSH import. Solute carrier family 25 member 39 (SLC25A39) is a mitochondrial membrane transporter required for GSH import, and its loss markedly reduces mitochondrial GSH levels (34). SLC25A39 expression is reduced in severe asthma. Functional studies in airway epithelial cells further show that reduced SLC25A39 is accompanied by decreased mitochondrial GSH and increased mtROS, whereas restoration of SLC25A39 increases mitochondrial GSH and attenuates these changes (35). Thus, increased GSH consumption during oxidized lipid reduction and insufficient mitochondrial GSH import can jointly limit mitochondrial detoxification of peroxides and oxidized membrane lipids.

Mitochondrial oxidative stress in asthma is characterized by an imbalance between ROS generation and antioxidant defense. Increased ROS generation can coexist with impaired superoxide clearance, constrained peroxide detoxification, and insufficient mitochondrial GSH availability. These changes limit the removal of excess ROS and oxidized membrane lipids. Persistent oxidative stress can further damage respiratory-chain proteins, antioxidant enzymes, membrane lipids, and other mitochondrial components, thereby promoting the accumulation of mitochondrial oxidative damage. The major abnormalities contributing to mitochondrial oxidative stress in asthma are summarized in **Figure 1**.

**Figure 1:**
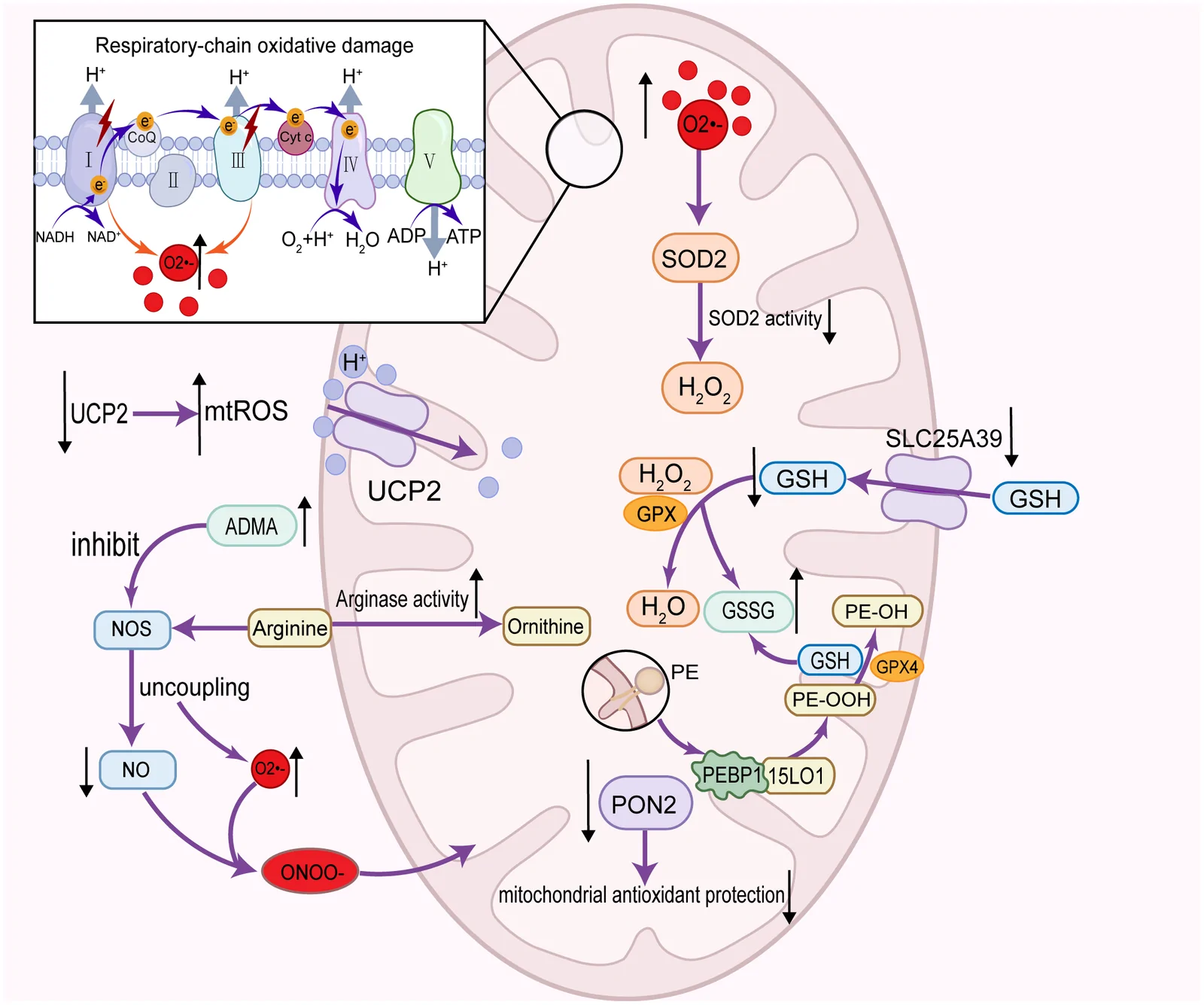
Mitochondrial oxidative stress in asthma. The mitochondrial respiratory chain is a source of superoxide, and oxidative damage to respiratory-chain components can alter ROS generation. Reduced UCP2 further increases mtROS. Enhanced arginase-mediated arginine metabolism and ADMA accumulation disrupt NOS–NO regulation, with NOS uncoupling accompanied by reduced NO bioavailability and increased superoxide and peroxynitrite formation. SOD2 converts superoxide to H_2_O_2_, whereas reduced SOD2 activity and PON2 levels weaken antioxidant defense. H_2_O_2_ is reduced in part through GSH-dependent GPX activity. The 15LO1–PEBP1 interaction promotes PE hydroperoxide formation, while GPX4 consumes GSH to reduce oxidized PE. Reduced SLC25A39 limits mitochondrial GSH import and decreases mitochondrial GSH availability. Together, increased ROS generation, impaired antioxidant defense, enhanced PE oxidation, and reduced mitochondrial GSH availability promote mitochondrial oxidative stress and oxidative damage. 15LO1, 15-lipoxygenase-1; ADMA, asymmetric dimethylarginine; ADP, adenosine diphosphate; ATP, adenosine triphosphate; CoQ, coenzyme Q; Cyt c, cytochrome c; GPX, glutathione peroxidase; GPX4, glutathione peroxidase 4; GSH, reduced glutathione; GSSG, oxidized glutathione; mtROS, mitochondrial reactive oxygen species; NAD^+^, oxidized nicotinamide adenine dinucleotide; NADH, reduced nicotinamide adenine dinucleotide; NO, nitric oxide; NOS, nitric oxide synthase; O2•^-^, superoxide; ONOO^-^, peroxynitrite; PE, phosphatidylethanolamine; PE-OOH, phosphatidylethanolamine hydroperoxide; PE-OH, hydroxy-phosphatidylethanolamine; PEBP1, phosphatidylethanolamine-binding protein 1; PON2, paraoxonase 2; SLC25A39, solute carrier family 25 member 39; SOD2, superoxide dismutase 2; UCP2, uncoupling protein 2.

### 2.2 Abnormalities in mitochondrial bioenergetics and substrate utilization

Mitochondrial bioenergetics depends on the coordinated processes of substrate entry and oxidation, the tricarboxylic acid (TCA) cycle, and oxidative phosphorylation (OXPHOS). Oxidation of glucose-derived pyruvate, fatty acids, and amino acids supplies carbon to the TCA cycle and generates reducing equivalents, including reduced nicotinamide adenine dinucleotide (NADH) and reduced flavin adenine dinucleotide (FADH_2_). These reducing equivalents donate electrons to the respiratory chain, and the resulting proton gradient drives ATP synthesis by ATP synthase (36, 37). Substrate entry and oxidation, the TCA cycle, and OXPHOS are functionally connected and together determine mitochondrial respiration and ATP production (38). Current evidence does not support a uniform pattern of impaired OXPHOS followed by compensatory glycolysis in asthma. Instead, abnormalities involve changes in substrate utilization together with altered relationships among mitochondrial respiration, ATP production, glycolysis, and TCA-related metabolism.

Mitochondrial respiration, ATP production, and glycolysis do not show a consistent direction of change in asthma. In mice with allergic airway inflammation, cytochrome c oxidase activity is reduced in lung mitochondria and is accompanied by lower pulmonary ATP levels (39). Peripheral blood mononuclear cells from patients with asthma show increased lactate production and glycolytic enzyme activity, together with reduced ATP production, respiratory-chain complex I and III activities, and citrate synthase activity. Similar metabolic changes are observed in the lungs of mice with ovalbumin (OVA)-induced allergic airway inflammation (40). These findings show that reduced oxidative energy metabolism can coexist with enhanced glycolysis in some asthma-associated settings. However, this pattern is not consistent across studies. Airway epithelial cells from patients with obesity-associated asthma show increased basal and maximal mitochondrial respiration together with enhanced glycolysis compared with those from patients with lean asthma and healthy controls (22). Peripheral blood mononuclear cells obtained during acute exacerbations of severe asthma also show increased basal respiration, adenosine diphosphate-stimulated respiration, and respiration supported by combined complex I and II substrates (41). In platelets from patients with asthma, ATP-linked respiration and proton leak are similar to those in healthy controls, whereas glycolysis is reduced and the activities of citrate synthase, aconitase, and succinate dehydrogenase are increased (42). Thus, mitochondrial respiration, ATP coupling, glycolysis, and TCA-cycle enzyme activity do not necessarily change in parallel. Enhanced glycolysis can accompany reduced oxidative metabolism in some settings, but the available evidence does not support a general compensatory glycolytic response to impaired OXPHOS in asthma.

Altered substrate utilization provides an additional explanation for these bioenergetic differences. One study reported lower ATP-linked oxygen consumption but greater spare respiratory capacity in platelets from patients with asthma, while patient-derived airway smooth muscle cells in the same study showed reduced mitochondrial glucose utilization (43). A separate study found increased fatty acid uptake and consumption in bronchial smooth muscle cells from patients with asthma, accompanied by enhanced mitochondrial fatty acid β-oxidation (FAO), mitochondrial respiration, and ATP production (44). These findings show that both glucose and fatty acid utilization can be altered in asthmatic airway smooth muscle. Because they were obtained in separate studies, they support altered mitochondrial substrate use rather than an established uniform switch from glucose to fatty acids.

Fatty acid metabolism in severe asthma also involves processes that determine the availability and mitochondrial entry of long-chain fatty acids. Urinary carnitine, acetylcarnitine, and propionylcarnitine are reduced in patients with severe asthma, together with lower enrichment of fatty acid metabolism-related pathways in sputum. Expression of the carnitine transporter solute carrier family 22 member 5 decreases with asthma severity in sputum and shows a similar downward trend in bronchial brushings (45). Carnitine palmitoyltransferase 1A (CPT1A) is also reduced in the airway epithelium of patients with severe asthma, and restoration of CPT1A expression increases mitochondrial respiration and ATP production (46). These findings suggest that fatty acid metabolic abnormalities in severe asthma can involve carnitine availability and transport as well as CPT1A-dependent entry of long-chain fatty acids into mitochondrial oxidative metabolism.

Carbon supply to the TCA cycle can also be altered through pathways other than glucose and fatty acid metabolism. Mitochondrial arginase 2 (ARG2) expression is increased in the airway epithelium of patients with asthma. ARG2-derived ornithine can be further converted to glutamate and α-ketoglutarate, providing a route for carbon entry into the TCA cycle. Increasing ARG2 expression in human bronchial epithelial cells increases mitochondrial oxygen consumption, glucose oxidation, and ATP production (47). These findings indicate that altered mitochondrial substrate use in asthma can also involve changes in the carbon sources entering the TCA cycle.

The available studies more consistently support altered mitochondrial substrate use together with non-parallel changes in respiration, ATP coupling, glycolysis, and TCA-related metabolism than a uniform reduction in mitochondrial energy production. Reduced oxidative metabolism accompanied by enhanced glycolysis occurs in some settings, whereas other studies show increased mitochondrial respiration, increased glycolysis, or reduced glycolysis. Glucose utilization and fatty acid utilization can also change, while reduced carnitine metabolism and CPT1A may constrain long-chain fatty acid entry into mitochondrial oxidative metabolism, and ARG2-related metabolism can provide additional carbon to the TCA cycle. The available evidence therefore does not define a uniform decrease in OXPHOS, a consistent compensatory increase in glycolysis, or a generalized alteration in TCA-cycle flux. Instead, asthma is associated with changes in the fuels available to mitochondria and their utilization, together with altered relationships among substrate oxidation, mitochondrial respiration, and ATP production. The major substrate-utilization pathways linking glucose, fatty acids, and ARG2-related carbon supply to mitochondrial respiration and ATP production are summarized in **Figure 2**.

**Figure 2:**
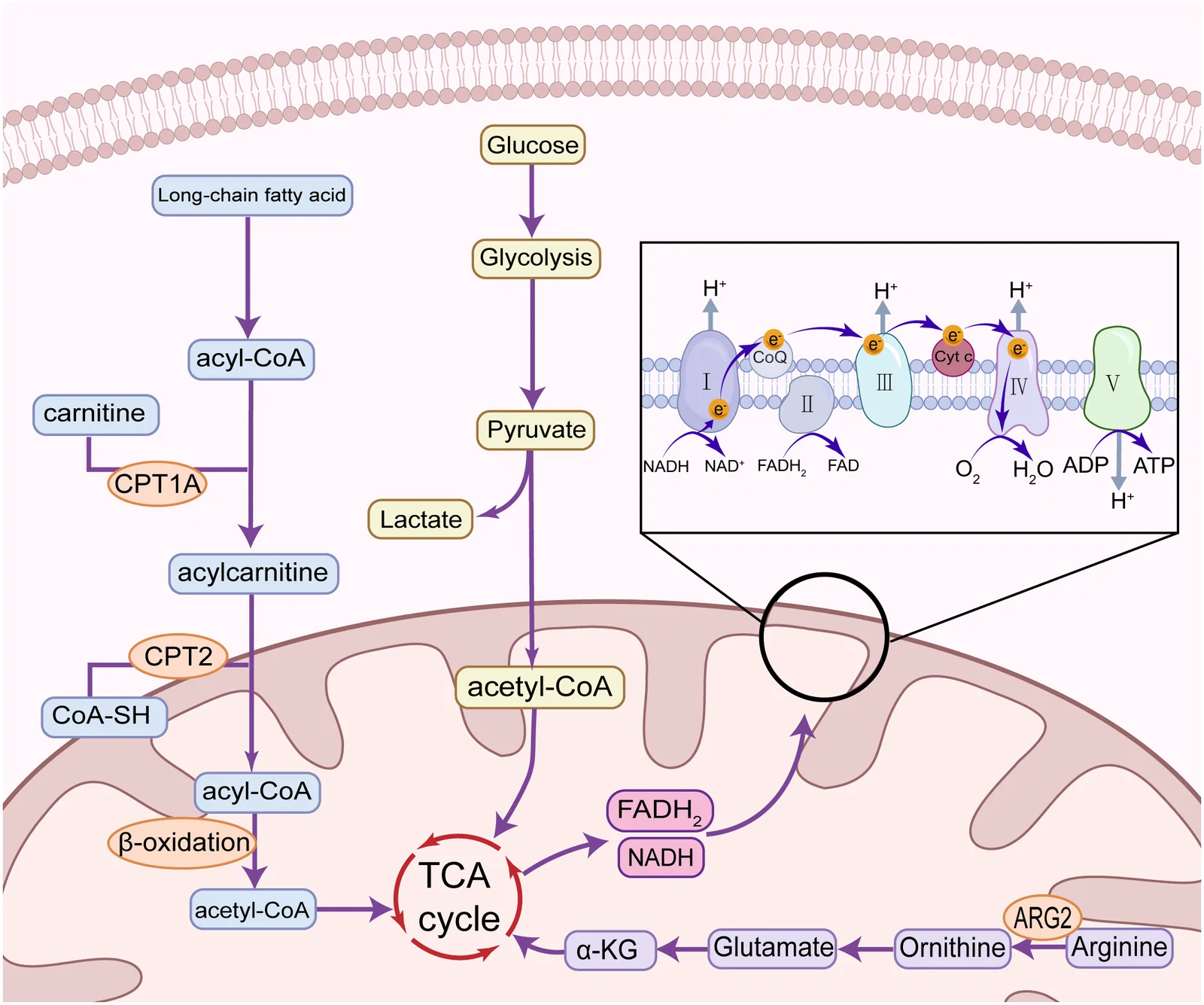
Mitochondrial bioenergetics and substrate utilization in asthma. Glucose-derived pyruvate can be converted to lactate or acetyl-CoA, with acetyl-CoA entering the TCA cycle. Long-chain fatty acids are activated to acyl-CoA and enter mitochondrial fatty acid oxidation through the carnitine shuttle involving CPT1A and CPT2, generating acetyl-CoA for the TCA cycle. ARG2-derived ornithine can be further converted through glutamate to α-KG, providing an additional carbon source for the TCA cycle. The TCA cycle generates NADH and FADH_2_, which donate electrons to the respiratory chain to establish the proton gradient that drives ATP synthesis. In asthma, changes in glucose and fatty acid utilization, carnitine-dependent fatty acid entry, and ARG2-related carbon supply alter the substrates available to the TCA cycle and are associated with changes in mitochondrial respiration and ATP production. α-KG, alpha-ketoglutarate; ADP, adenosine diphosphate; ARG2, arginase 2; ATP, adenosine triphosphate; CoA, coenzyme A; CoQ, coenzyme Q; CPT1A, carnitine palmitoyltransferase 1A; CPT2, carnitine palmitoyltransferase 2; Cyt c, cytochrome c; FAD, flavin adenine dinucleotide; FADH_2_, reduced flavin adenine dinucleotide; NAD^+^, oxidized nicotinamide adenine dinucleotide; NADH, reduced nicotinamide adenine dinucleotide; TCA, tricarboxylic acid.

### 2.3 Abnormalities in mitochondrial Ca^2+^ homeostasis

Following Ca^2+^ release from the endoplasmic or sarcoplasmic reticulum, local regions of high Ca^2+^ concentration can form near the release sites, allowing adjacent mitochondria to take up Ca^2+^. At endoplasmic reticulum–mitochondria contact sites, glucose-regulated protein 75 (GRP75) links an endoplasmic reticulum Ca^2+^ release channel to a channel in the outer mitochondrial membrane, facilitating Ca^2+^ transfer toward mitochondria (48). Ca^2+^ subsequently enters the mitochondrial matrix across the inner membrane through the mitochondrial calcium uniporter (MCU), a process that also contributes to the regulation of aerobic metabolism (49), and can be extruded from mitochondria through the mitochondrial Na^+^/Ca^2+^ exchanger (NCLX) (50). Mitochondrial Ca^2+^ levels are therefore jointly regulated by cytosolic Ca^2+^ load, Ca^2+^ transfer from the endoplasmic or sarcoplasmic reticulum, and mitochondrial Ca^2+^ uptake and efflux. Asthma-related studies indicate that alterations can occur at several of these steps, resulting in different patterns of cytosolic and mitochondrial Ca^2+^ distribution.

Changes in cytosolic Ca^2+^ levels and in Ca^2+^ storage and reuptake by the endoplasmic or sarcoplasmic reticulum can alter mitochondrial Ca^2+^ load. The linoleic acid oxidation product 13(S)-hydroxyoctadecadienoic acid, which is increased in asthma, increases intracellular and mitochondrial Ca^2+^ levels in human bronchial epithelial cells (51). In airway smooth muscle cells, sarco/endoplasmic reticulum Ca^2+^-ATPase 2 (SERCA2) pumps cytosolic Ca²^+^ back into the sarcoplasmic reticulum. Airway smooth muscle cells from patients with asthma have been reported to exhibit reduced SERCA2 expression, impaired replenishment of sarcoplasmic reticulum Ca^2+^ stores, and delayed recovery of cytosolic Ca^2+^, whereas another study using patient-derived cells found no significant changes in SERCA2 expression or Ca^2+^ handling (52, 53). Exposure of human airway smooth muscle cells to tumor necrosis factor alpha (TNF-α) and IL-13 can also reduce SERCA2 expression and slow Ca^2+^ reuptake (54). These findings indicate that SERCA2 alterations are not consistent across studies of asthmatic airway smooth muscle and may vary with inflammatory conditions and experimental settings. Disrupted phospholipid synthesis in airway epithelial cells can also disturb endoplasmic reticulum Ca^2+^ homeostasis and is accompanied by mitochondrial Ca^2+^ overload (55). Changes in cytosolic Ca^2+^ and in endoplasmic or sarcoplasmic reticulum Ca^2+^ storage and reuptake can therefore alter the Ca^2+^ environment to which mitochondria are exposed.

Ca^2+^ transfer between the endoplasmic reticulum and mitochondria, together with MCU-mediated uptake, further determines the amount of Ca^2+^ entering the mitochondrial matrix. In Aspergillus fumigatus-induced allergic airway inflammation, endoplasmic reticulum–mitochondria contacts are increased and are accompanied by greater mitochondrial Ca^2+^ accumulation (56). High mobility group box 1 induces GRP75 upregulation and promotes Ca^2+^ transfer from the endoplasmic reticulum to mitochondria (57). IL-13-induced mitochondrial Ca^2+^ uptake is dependent on MCU, and MCU inhibition markedly reduces mitochondrial Ca^2+^ uptake (58). These findings indicate that enhanced endoplasmic reticulum–mitochondria contact and Ca^2+^ transfer, together with increased MCU-mediated uptake, can promote Ca^2+^ accumulation within the mitochondrial matrix.

Mitochondrial Ca^2+^ dysregulation can also involve reduced local uptake or abnormal Ca^2+^ efflux. In human airway smooth muscle cells, MCU inhibition enhances stimulus-induced cytosolic Ca²^+^ responses. Under TNF-α exposure, cytosolic Ca^2+^ responses increase while Ca^2+^ responses in perinuclear mitochondria decrease, whereas mitochondria located farther from the nucleus do not show the same change (59). Reduced mitochondrial Ca^2+^ uptake or locally blunted mitochondrial Ca^2+^ responses can therefore coexist with enhanced cytosolic Ca^2+^ signaling. Physiological mitochondrial Ca^2+^ uptake contributes to the regulation of aerobic metabolism, and reduced uptake may therefore affect the coupling between cytosolic Ca^2+^ signals and mitochondrial metabolism, although this effect has not been directly demonstrated in asthma-related airway cells. Excessive Ca^2+^ transfer and uptake and insufficient mitochondrial Ca^2+^ uptake can produce different consequences. The former promotes Ca^2+^ accumulation within the mitochondrial matrix, whereas the latter can reduce mitochondrial buffering of cytosolic Ca^2+^ signals and may alter the coupling between Ca^2+^ signaling and mitochondrial metabolism. Abnormal Ca^2+^ efflux produces another pattern. In human airway smooth muscle cells, reduced NCLX increases mitochondrial Ca^2+^ retention while decreasing store-operated Ca^2+^ entry (60). These findings indicate that mitochondrial Ca^2+^ levels and cytosolic Ca^2+^ signaling do not necessarily change in parallel.

Mitochondrial Ca^2+^ homeostasis in asthma can therefore be altered at multiple points that regulate Ca^2+^ distribution between the cytosol and mitochondria. Cytosolic Ca^2+^ levels, Ca^2+^ storage and reuptake by the endoplasmic or sarcoplasmic reticulum, endoplasmic reticulum–mitochondria Ca^2+^ transfer, MCU-mediated uptake, and NCLX-mediated efflux can all be affected. Enhanced Ca^2+^ transfer and uptake can promote Ca^2+^ accumulation within the mitochondrial matrix, whereas reduced mitochondrial uptake or locally blunted mitochondrial Ca^2+^ responses can be accompanied by enhanced cytosolic Ca^2+^ signaling and may weaken its coupling to mitochondrial metabolism. Restricted NCLX-mediated efflux can instead increase mitochondrial Ca^2+^ retention while altering cytosolic Ca^2+^ entry. Mitochondrial Ca^2+^ dysregulation in asthma is therefore better characterized as an imbalance in Ca^2+^ distribution and transport, with the direction of change depending on the specific Ca^2+^ regulatory step affected. The principal Ca^2+^ transport processes underlying this imbalance in Ca^2+^ distribution among the cytosol, endoplasmic or sarcoplasmic reticulum, and mitochondria are summarized in **Figure 3**.

**Figure 3:**
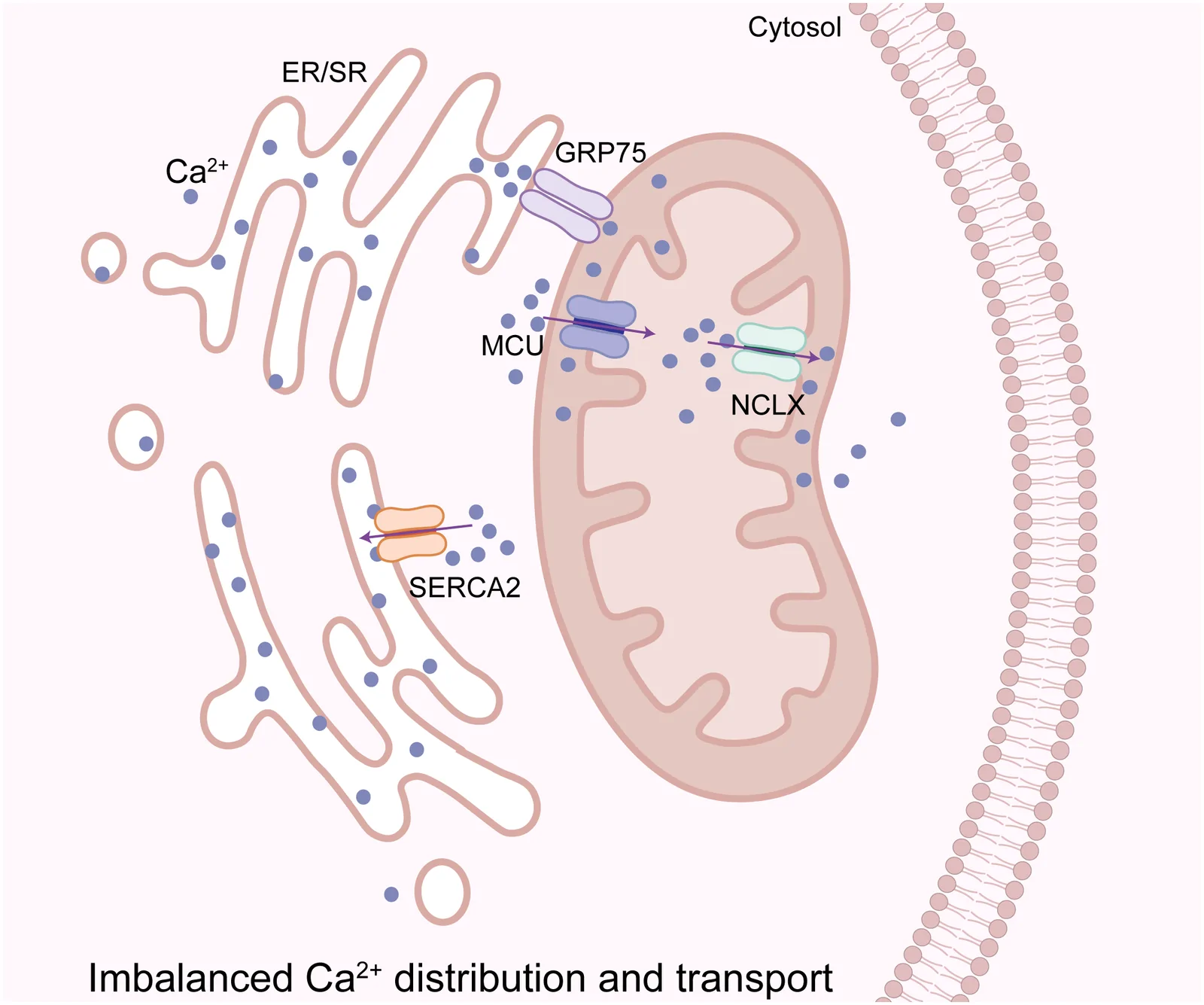
Mitochondrial Ca^2+^ distribution and transport abnormalities in asthma. Ca^2+^ released from the endoplasmic or sarcoplasmic reticulum can be transferred toward mitochondria at contact sites facilitated by GRP75 and subsequently enter the mitochondrial matrix through MCU. NCLX mediates mitochondrial Ca^2+^ efflux, whereas SERCA2 returns cytosolic Ca^2+^ to the endoplasmic or sarcoplasmic reticulum. In asthma-related studies, ER/SR Ca^2+^ storage and reuptake, ER/SR–mitochondria Ca^2+^ transfer, MCU-dependent uptake, local mitochondrial Ca^2+^ responses, and NCLX-mediated efflux can all be altered. Enhanced ER/SR–mitochondria Ca^2+^ transfer and mitochondrial uptake can promote mitochondrial Ca^2+^ accumulation, whereas reduced local mitochondrial Ca^2+^ uptake can coexist with enhanced cytosolic Ca^2+^ signaling. Restricted NCLX-mediated efflux increases mitochondrial Ca^2+^ retention. These changes disturb the distribution of Ca^2+^ among the cytosol, ER/SR, and mitochondria. ER, endoplasmic reticulum; GRP75, glucose-regulated protein 75; MCU, mitochondrial calcium uniporter; NCLX, mitochondrial Na^+^/Ca²^+^ exchanger; SERCA2, sarco/endoplasmic reticulum Ca^2+^-ATPase 2; SR, sarcoplasmic reticulum.

### 2.4 Abnormalities in mitochondrial dynamics

Mitochondria continuously adjust their morphology and intracellular distribution through fission, fusion, and intracellular transport (61). Dynamin-related protein 1 (DRP1) promotes mitochondrial fission after being recruited from the cytosol to the outer mitochondrial membrane (62). Mitofusin 1 and 2 (MFN1 and MFN2) mediate outer membrane fusion (63), whereas optic atrophy 1 (OPA1) mediates inner membrane fusion (64). Miro and Milton/TRAK-related proteins participate in mitochondrial motility and intracellular distribution (65, 66). Mitochondrial dynamics abnormalities in asthma can involve shortened or fragmented mitochondrial morphology, dysregulated fission–fusion regulation, and impaired mitochondrial motility and positioning.

Shortened and fragmented mitochondrial morphology is frequently reported in asthma-related studies. Airway smooth muscle cells from patients with asthma exhibit lower mitochondrial form factor and aspect ratio than cells from individuals without asthma (67). Asthma-related stimulation of airway epithelial cells can also induce DRP1 activation and increased mitochondrial fission, accompanied by loss of mitochondrial network integrity (68). In human airway smooth muscle cells, inflammatory stimulation can concurrently increase DRP1, reduce MFN2, and enhance mitochondrial fragmentation (69). These findings associate asthma-related mitochondrial morphological alterations with enhanced regulation of mitochondrial fission.

DRP1 abnormalities can occur at several regulatory steps, including protein abundance, phosphorylation, mitochondrial recruitment, and protein assembly. Restoration of microRNAs involved in DRP1 regulation reduces DRP1 expression or pro-fission regulation and is accompanied by recovery of MFN1 or MFN2 and reduced mitochondrial fragmentation (70, 71). Other asthma-related studies have reported increased pro-fission phosphorylation of DRP1 at Ser616 and enhanced recruitment of DRP1 to mitochondria. Interventions targeting the corresponding upstream regulators reduce DRP1 activation and mitochondrial fission (72–75). Increased DRP1 oligomerization has also been observed in airway myeloid-derived regulatory cells in asthma (76). DRP1 dysregulation in asthma therefore extends beyond changes in protein abundance to enhanced activation, mitochondrial recruitment, and oligomerization, promoting mitochondrial fission through several regulatory steps.

Fusion-related regulation can also be altered. Reduction of stromal interaction molecule 1 increases MFN2 and OPA1 expression and is accompanied by enhanced mitochondrial fusion (77). In house dust mite (HDM)-induced asthmatic mice and airway epithelial cells, attenuation of mitochondrial injury is likewise accompanied by increased MFN2 expression and reduced mitochondrial fission (78). These findings indicate that fusion-related regulation can act together with changes in fission to influence mitochondrial network organization. At the same time, maintenance of mitochondrial homeostasis requires an appropriate level of fission. Although airway club cell-specific deletion of *Drp1* reduces mitochondrial fission, it aggravates epithelial cell death, airway inflammation, and airway hyperresponsiveness (79). Mitochondrial dynamics abnormalities in asthma are therefore better characterized by dysregulation of the balance between fission and fusion than by the degree of fission alone.

Mitochondrial motility and intracellular positioning can also be affected. Following TNF-α exposure in human airway smooth muscle cells, Miro2 and Milton2 levels decrease, accompanied by reductions in the number of motile mitochondria and the velocity of directional mitochondrial movement, together with an increased distance between mitochondria and sarcoplasmic reticulum Ca^2+^ release regions (80). Loss of Miro1 in airway club cells causes marked redistribution of mitochondria within the cell (81). These findings indicate that mitochondrial dynamics abnormalities in asthma involve not only mitochondrial network morphology and fission–fusion regulation but also mitochondrial movement and spatial distribution within the cell.

Asthma-related abnormalities in mitochondrial dynamics involve shortening and fragmentation of the mitochondrial network, enhanced DRP1-related fission regulation, alterations in fusion-related regulation, and impaired mitochondrial motility and positioning. DRP1 can be dysregulated at multiple levels, including protein abundance, phosphorylation, mitochondrial recruitment, and oligomerization, while an appropriate balance between fission and fusion remains necessary for mitochondrial homeostasis. Mitochondrial dynamics abnormalities in asthma are therefore better characterized as a combined disturbance of mitochondrial morphology, fission–fusion regulation, and intracellular distribution rather than simply as increased mitochondrial fission. The principal alterations in mitochondrial fission–fusion regulation, motility, and intracellular distribution in asthma are summarized in **Figure 4**.

**Figure 4:**
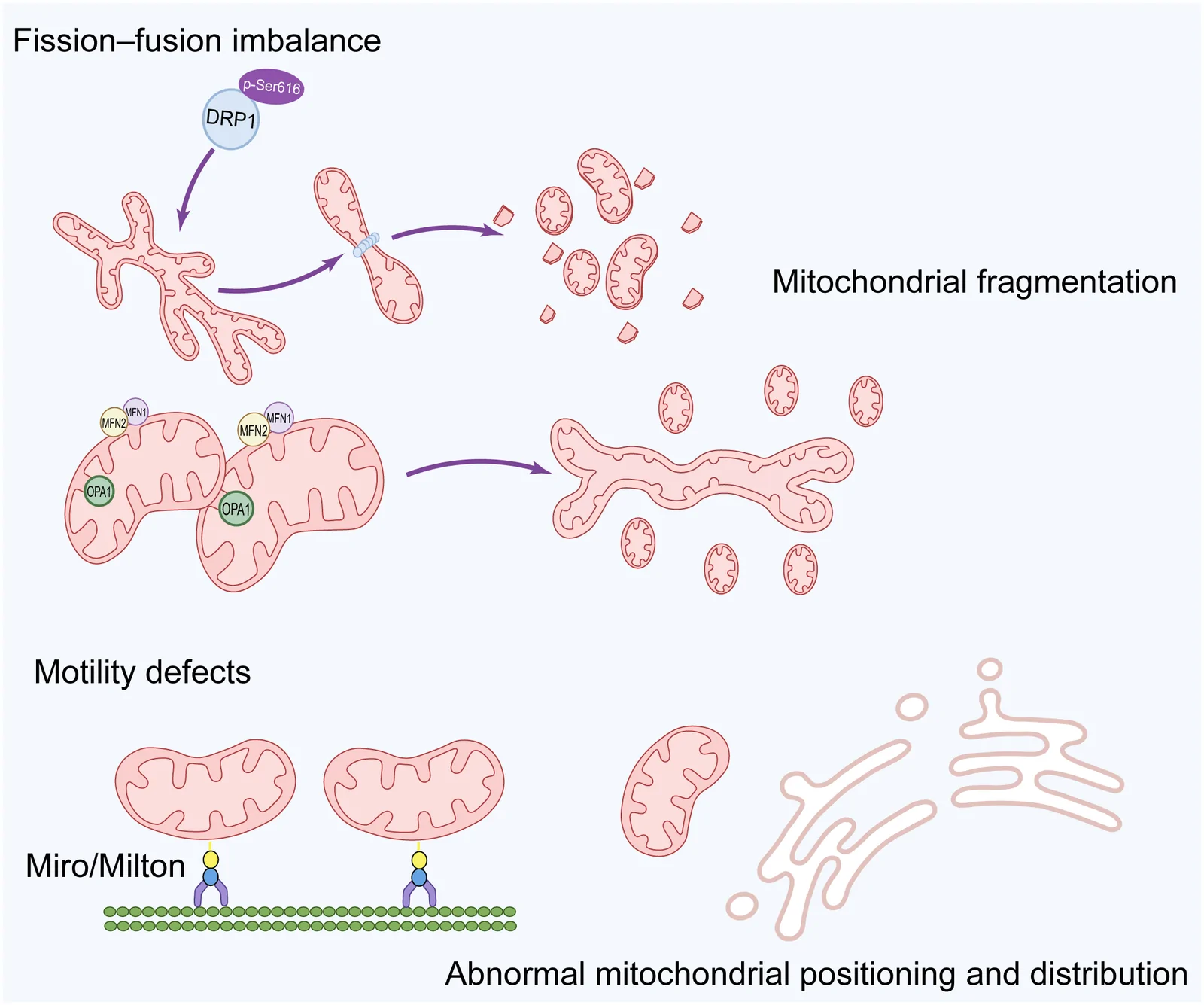
Mitochondrial dynamics abnormalities in asthma. DRP1 is recruited to the outer mitochondrial membrane to promote mitochondrial fission, and pro-fission phosphorylation of DRP1 at Ser616 can further enhance this process. MFN1 and MFN2 mediate outer mitochondrial membrane fusion, whereas OPA1 mediates inner membrane fusion. Altered fission–fusion regulation is associated with mitochondrial shortening, fragmentation, and loss of mitochondrial network integrity in asthma-related studies. Miro- and Milton-related proteins participate in mitochondrial motility and intracellular positioning, and abnormalities in these processes can reduce mitochondrial movement and alter mitochondrial positioning and distribution within the cell. Together, disturbances in fission–fusion regulation, mitochondrial motility, and intracellular positioning contribute to abnormal mitochondrial dynamics in asthma. DRP1, dynamin-related protein 1; MFN1, mitofusin 1; MFN2, mitofusin 2; OPA1, optic atrophy 1.

### 2.5 Abnormalities in mitochondrial biogenesis

Mitochondrial biogenesis is the process by which mitochondrial mass and number increase through the growth and division of pre-existing mitochondria and requires coordinated synthesis of components encoded by the nuclear genome and mtDNA (82). Peroxisome proliferator-activated receptor gamma coactivator 1-alpha (PGC-1α) cooperates with transcription factors such as nuclear respiratory factor 1 (NRF1) to promote the expression of mitochondrial transcription factor A (TFAM) and other nuclear-encoded mitochondrial genes. TFAM participates in mtDNA transcription and regulates mtDNA copy number (83, 84). Asthma-related studies have reported findings consistent with both enhanced and reduced mitochondrial biogenesis, and changes in biogenesis-related factors do not always parallel changes in mitochondrial abundance.

Evidence consistent with enhanced mitochondrial biogenesis includes increased PGC-1α–NRF1–TFAM-related regulation together with increased mitochondrial abundance. PGC-1α, NRF1, and TFAM are increased in bronchial smooth muscle from patients with asthma, accompanied by increased mitochondrial content (85). Increased mitochondrial number and density have also been observed in bronchial smooth muscle from some patients with non-severe asthma (86). Altered upstream regulation of PGC-1α can further increase biogenesis-related signaling. In bronchial smooth muscle cells from individuals without asthma, p53 suppresses the transcription of PGC-1α and TFAM, whereas this repression is attenuated in cells from patients with asthma (87). In primary human airway smooth muscle cells, immunoglobulin E (IgE)-associated phosphoinositide 3-kinase (PI3K)–protein kinase B (Akt) signaling increases PGC-1α expression (88). In lung fibroblasts, protein arginine methyltransferase 1 (PRMT1) and PGC-1α are increased in asthma-derived cells, whereas PRMT1 inhibition reduces PGC-1α expression and attenuates the TGF-β-induced increase in mitochondrial content (89). Together, these findings indicate that enhanced PGC-1α-related regulation of mitochondrial biogenesis can result from attenuated transcriptional repression or increased upstream signaling that promotes PGC-1α expression.

Biogenesis-related regulation and total mitochondrial abundance can also diverge. In bronchial fibroblasts from patients with severe asthma, sirtuin 1 (SIRT1) and PGC-1α are increased, whereas mitochondrial content does not differ significantly from that in healthy control cells and clearance of damaged mitochondria is enhanced (90). This pattern indicates that increased biogenesis-related regulation can coexist with enhanced clearance of damaged mitochondria while total mitochondrial abundance remains relatively stable. In bronchial fibroblasts from patients with severe asthma, IL-17 exposure reduces the elevated levels of SIRT1 and PGC-1α and is accompanied by reduced mitochondrial content (91), further indicating that biogenesis-related regulation can vary with the inflammatory environment.

Findings consistent with reduced mitochondrial biogenesis have also been reported in airway epithelial cells. Following HDM exposure, human bronchial epithelial cells show concurrent reductions in PGC-1α, TFAM, and mitochondrial content. Enhancing PGC-1α-related mitochondrial biogenesis restores TFAM and mitochondrial content, while reduced PGC-1α and TFAM are also observed in the bronchial epithelium of HDM-induced asthmatic mice (92). In IL-4-associated mitochondrial injury in airway epithelial cells, ADMA accumulation is accompanied by reduced PGC-1α and TFAM expression and mitochondrial loss, whereas increasing TFAM expression partially restores mitochondrial abundance and morphology (23). These findings indicate that reduced PGC-1α–TFAM-related regulation of mitochondrial biogenesis can accompany mitochondrial loss in asthma-related airway epithelium.

Mitochondrial biogenesis in asthma can therefore be enhanced or reduced, and the direction of biogenesis-related regulation does not by itself determine total mitochondrial abundance. Enhanced PGC-1α-related regulation can accompany increased mitochondrial abundance or coexist with enhanced clearance of damaged mitochondria while mitochondrial abundance remains relatively stable, whereas reduced PGC-1α–TFAM-related regulation can accompany mitochondrial loss. Assessment of mitochondrial biogenesis in asthma should therefore integrate changes in PGC-1α, NRF1, TFAM, and related regulators with mitochondrial abundance and concurrent mitochondrial turnover. The relationship between mitochondrial biogenesis-related regulation, mitochondrial abundance, and mitochondrial turnover in asthma is summarized in **Figure 5**.

**Figure 5:**
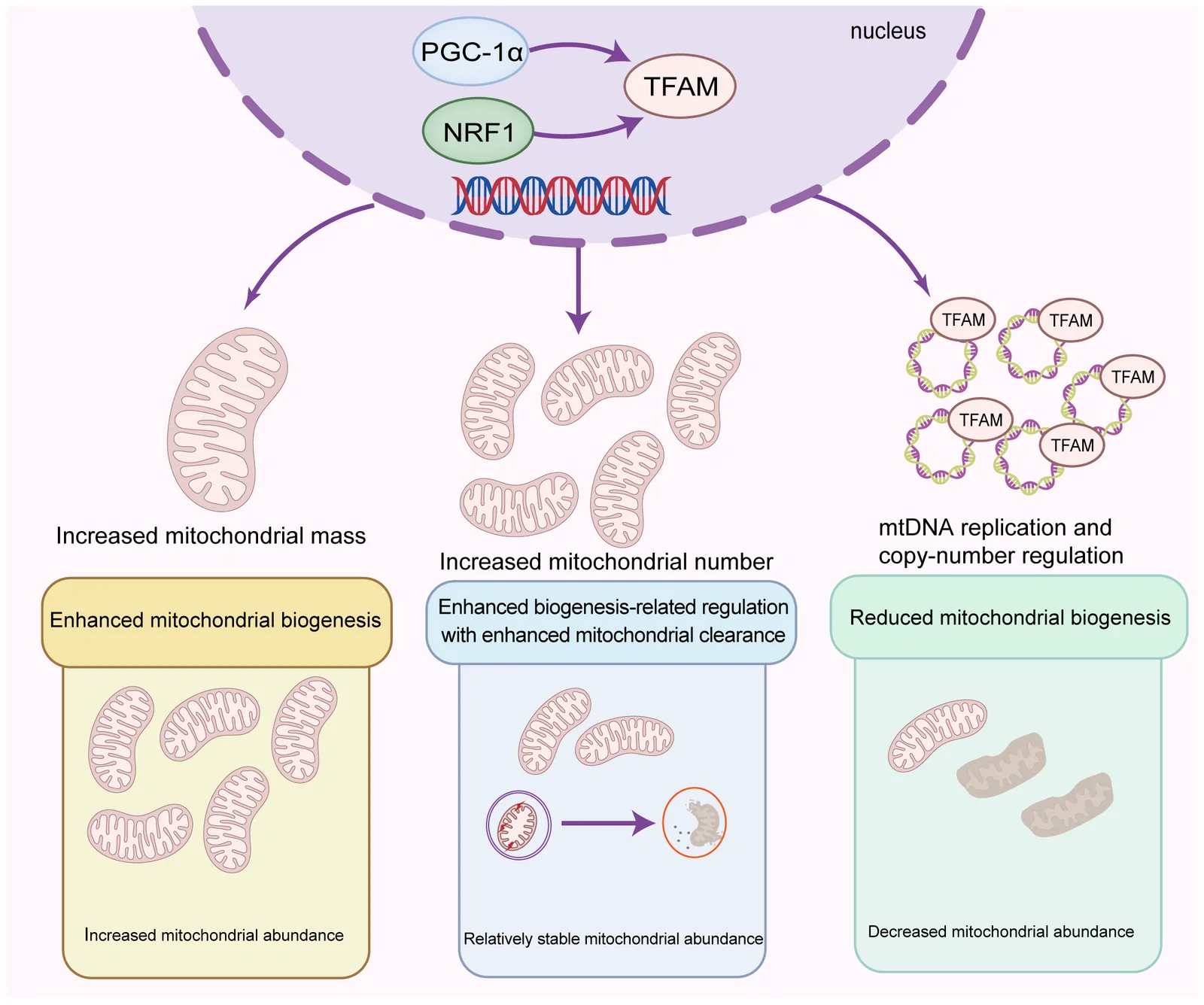
Mitochondrial biogenesis and mitochondrial abundance in asthma. PGC-1α cooperates with NRF1 to regulate TFAM and other nuclear-encoded mitochondrial genes involved in mitochondrial biogenesis. TFAM participates in mtDNA transcription and copy-number regulation, while mitochondrial biogenesis increases mitochondrial mass and number through the growth and division of pre-existing mitochondria. In asthma, enhanced biogenesis-related regulation can accompany increased mitochondrial abundance, whereas reduced PGC-1α–TFAM-related regulation can accompany mitochondrial loss. Increased biogenesis-related regulation can also coexist with enhanced clearance of damaged mitochondria, allowing total mitochondrial abundance to remain relatively stable. Mitochondrial abundance therefore reflects the balance between mitochondrial biogenesis-related regulation and mitochondrial turnover. mtDNA, mitochondrial DNA; NRF1, nuclear respiratory factor 1; PGC-1α, peroxisome proliferator-activated receptor gamma coactivator 1-alpha; TFAM, mitochondrial transcription factor A.

### 2.6 Abnormalities in mitophagy

Mitophagy maintains mitochondrial quality homeostasis through the selective removal of damaged mitochondria (93). In PTEN-induced kinase 1 (PINK1)/Parkin-dependent mitophagy, PINK1 accumulates on damaged mitochondria and activates Parkin, promoting ubiquitination of outer mitochondrial membrane proteins. Selective autophagy receptors such as optineurin (OPTN) subsequently recognize ubiquitinated mitochondria and interact with microtubule-associated protein 1 light chain 3 (LC3), allowing damaged mitochondria to be sequestered into autophagosomes and ultimately degraded by lysosomes (94, 95). Mitochondrial outer membrane receptors such as BCL2-interacting protein 3 (BNIP3) and BCL2-interacting protein 3-like (BNIP3L/NIX) can also bind directly to LC3 and mediate receptor-dependent mitophagy independently of PINK1/Parkin (96). Mitophagy abnormalities in asthma mainly involve the capacity to remove damaged mitochondria and the coordination of successive clearance steps. In some settings, the entire process from damage recognition to final degradation is enhanced, whereas in others these steps change discordantly.

Several asthma-related studies support an overall increase in damaged mitochondrial clearance. In bronchial fibroblasts from patients with severe asthma, PINK1 and Parkin are increased together with mitophagy-related proteins such as BNIP3 and BNIP3L/NIX. Entry of damaged mitochondria into autophagic and lysosomal degradation is also increased, and flux assays further support enhanced mitophagy (90). Airway epithelial studies further show that PINK1/Parkin-mediated damage recognition and OPTN-dependent recruitment of the autophagic machinery can jointly promote this process. Following IL-13 exposure, PINK1 expression and Parkin phosphorylation increase, while OPTN–LC3 interaction is enhanced and accompanied by increased mitophagy. OPTN is also increased in the airway epithelium of patients with asthma characterized by higher type 2 inflammation, whereas other mitophagy receptors examined in the same study do not show consistent changes (33). In primary human bronchial epithelial cells exposed to asthma-related stimuli, OPTN recruitment to mitochondria increases, whereas OPTN reduction decreases mitophagy (97). Other intervention studies likewise show that modulation of mitophagy-related regulatory processes can alter mitophagy levels (74, 98). Together, these findings support a coordinated increase in mitochondrial damage recognition, recruitment of the autophagic machinery, and subsequent clearance in some asthma-related settings, with relatively direct functional evidence for the involvement of OPTN.

Different stages of mitochondrial clearance can also change discordantly. Following IL-17 exposure of bronchial fibroblasts from patients with severe asthma, markers of general autophagy increase, whereas PINK1 and Parkin protein levels decrease despite increased corresponding transcript levels (91), indicating that increased general autophagy does not always parallel PINK1/Parkin-associated mitophagy. Thymic stromal lymphopoietin (TSLP) exposure also produces different patterns across experimental systems. Mitophagy signals are reduced in a human bronchial epithelial cell line and in blood monocytes from patients with asthma after TSLP exposure (99), whereas another monocyte system shows increased mitochondrial accumulation of PINK1, phosphorylated Parkin, and LC3 following TSLP exposure (100). Changes in PINK1, Parkin, or LC3 have also been observed following IL-25 or IL-33 exposure (101, 102). These findings indicate that increases in markers related to damage recognition or recruitment of the autophagic machinery mainly reflect upstream stages of mitophagy. Completion of damaged mitochondrial clearance additionally depends on subsequent autophagosomal processing and lysosomal degradation. Changes in PINK1, Parkin, or LC3 alone are therefore insufficient to establish increased mitophagic flux.

Mitophagy abnormalities in asthma are therefore mainly characterized by changes in damaged mitochondrial clearance and in the coordination of the clearance process. In some settings, damage recognition, recruitment of the autophagic machinery, and lysosomal degradation are all enhanced, resulting in increased removal of damaged mitochondria. In others, upstream mitophagy-related changes are not matched by subsequent degradation. Assessment of mitophagy in asthma should therefore integrate mitochondrial damage recognition, recruitment of the autophagic machinery, and final lysosomal degradation, whereas individual markers such as PINK1, Parkin, and LC3 mainly reflect specific stages of this process. The principal steps involved in damaged mitochondrial recognition, autophagic recruitment, and lysosomal clearance in asthma-related mitophagy are summarized in **Figure 6**.

**Figure 6:**
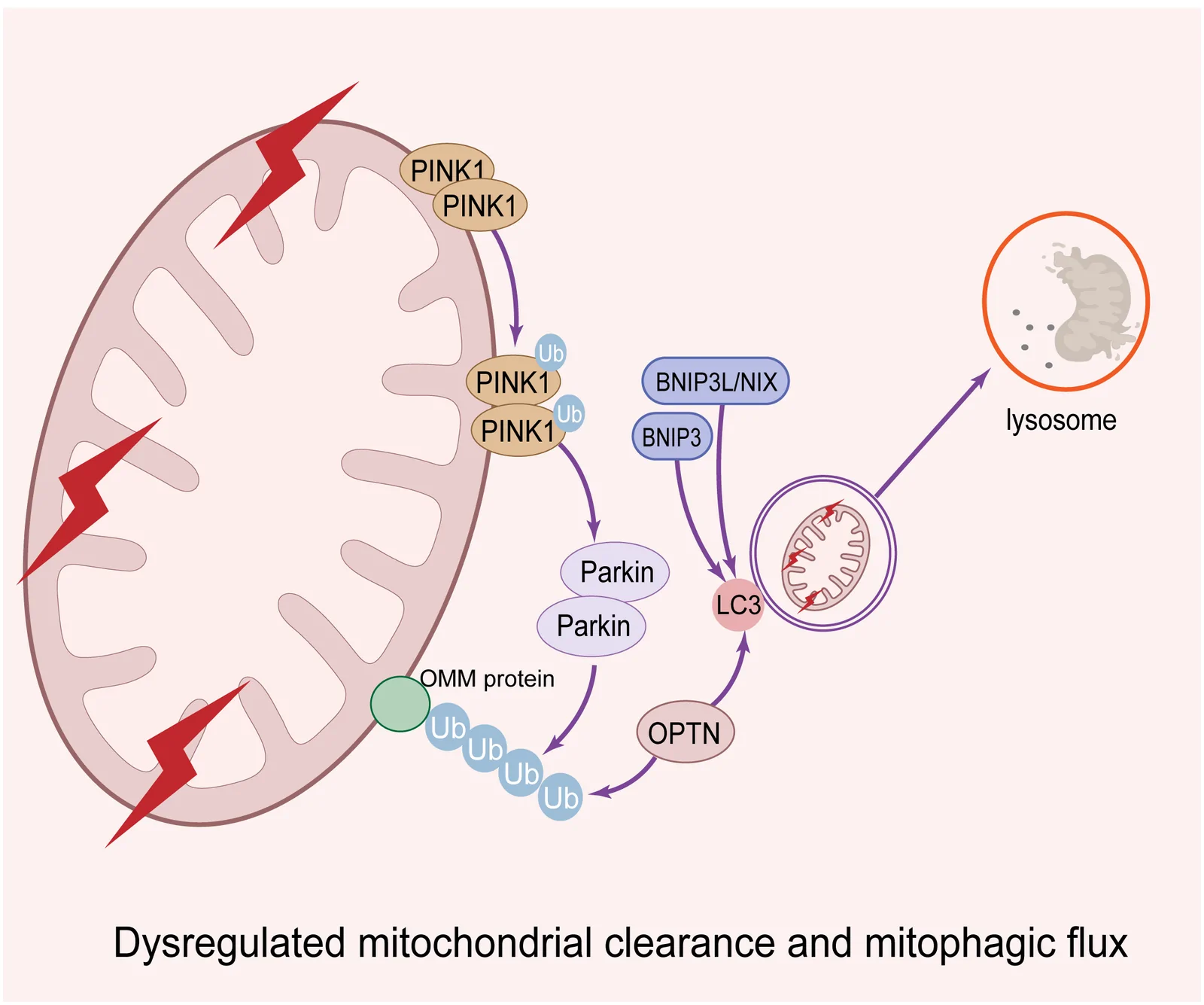
Mitophagy and mitochondrial clearance abnormalities in asthma. PINK1 accumulates on damaged mitochondria and activates Parkin, which promotes ubiquitination of outer mitochondrial membrane proteins. OPTN recognizes ubiquitinated mitochondria and interacts with LC3, facilitating sequestration of damaged mitochondria into autophagosomes and subsequent lysosomal degradation. BNIP3 and BNIP3L/NIX can also interact directly with LC3 and mediate receptor-dependent mitophagy independently of PINK1/Parkin. In asthma, damage recognition, autophagic recruitment, and lysosomal degradation can show coordinated increases in some settings, whereas changes in upstream mitophagy-related markers are discordant with later clearance steps in others. Alterations in PINK1, Parkin, or LC3 alone therefore do not establish increased mitophagic flux. BNIP3, BCL2-interacting protein 3; BNIP3L/NIX, BCL2-interacting protein 3-like/NIX; LC3, microtubule-associated protein 1 light chain 3; OMM, outer mitochondrial membrane; OPTN, optineurin; PINK1, PTEN-induced kinase 1; Ub, ubiquitin.

### 2.7 Heterogeneity of mitochondrial dysfunction in asthma

Current evidence indicates that mitochondrial abnormalities differ across asthma endotypes and phenotypes. Among type 2 inflammatory endotypes, transcriptomic alterations in the airway epithelium of patients with type 2-low asthma mainly involve mitochondrial ATP synthesis-coupled electron transport, respiratory-chain complex assembly, and respiratory electron transport (103). In type 2-high asthma, lipid peroxidation, autophagic responses, and changes in mtDNA release have also been reported. In the airway epithelium of patients with type 2-high severe asthma, 15LO1–PEBP1-associated phospholipid oxidation occurs together with enhanced autophagic responses and is linked to changes in mtDNA release (104). Current evidence therefore suggests that mitochondrial alterations in type 2-low asthma are mainly reflected in transcriptional changes related to the respiratory chain and electron transport, whereas type 2-high asthma involves lipid peroxidation, autophagic responses, and changes in mtDNA release.

Different metabolic and mitochondrial transcriptional features have also been observed across airway inflammatory phenotypes. Transcriptomic analysis of induced sputum showed enrichment of OXPHOS-related gene sets in eosinophilic asthma, whereas Toll-like receptor and NOD-like receptor signaling pathways were predominantly enriched in neutrophilic asthma (105). Mitochondria-related gene expression also differs between neutrophilic and non-neutrophilic asthma (106). These findings indicate that eosinophilic and neutrophilic asthma exhibit distinct features in OXPHOS-related transcriptional programs and mitochondria-related gene expression.

Clinical phenotypes and disease severity are also associated with different mitochondrial alterations. Airway epithelial cells from patients with obesity-associated asthma exhibit distinct mitochondrial bioenergetic features compared with those from patients with lean asthma (22). Differences according to disease severity involve mitochondrial energy metabolism, substrate utilization, and structure. Comparisons of bronchial epithelial cells across different levels of asthma severity showed that reduced expression of OXPHOS-related genes was more pronounced in severe asthma, whereas changes in mild asthma were smaller and more heterogeneous among patients (107). Induced-sputum transcriptomes from patients with severe asthma also showed lower mitochondria-related gene activity, with associated changes involving OXPHOS, cellular respiration, and ROS-related processes; a mitochondria-related 9-gene signature derived from these analyses was further validated in an independent patient dataset (108). Severe asthma is also associated with abnormalities in carnitine and fatty acid metabolism, consistent with alterations in metabolic processes involved in mitochondrial fatty acid utilization (45). At the structural level, mitochondrial damage in the bronchial epithelium is more pronounced in patients with severe asthma than in those with mild asthma and healthy controls (109). However, mitochondrial measures do not change consistently with disease severity. Blood mtDNA copy number is lower in individuals with asthma than in those without asthma, but no clear differences according to asthma severity were observed in either the UK Biobank or the Severe Asthma Research Program cohort (SARP); in the longitudinal SARP cohort, higher mtDNA copy number was associated with a lower risk of subsequent exacerbations (110).

In addition to differences across asthma endotypes and phenotypes, variation in upstream stimuli represents another source of heterogeneity in mitochondrial abnormalities in asthma. Different stimuli can affect distinct aspects of mitochondrial function, while a single stimulus may perturb multiple mitochondrial processes. The major upstream factors investigated to date include inhaled allergens and other biological stimuli, environmental and occupational exposures, inflammatory and immune signals, and metabolic abnormalities and other endogenous factors. Inhaled allergens and other biological stimuli, including house dust mite, ragweed pollen, cockroach allergen, and fungal stimuli, have been linked to mitochondrial oxidative injury, altered mitochondrial biogenesis, and changes in mitophagy-related regulation (20, 21, 92, 97). Environmental and occupational exposures include particulate pollutants such as fine particulate matter (PM2.5), oxidizing gases such as ozone, combustion-related exposures such as cigarette smoke and diesel exhaust particles, and occupational chemicals such as toluene diisocyanate. PM2.5 exposure can promote DRP1-related mitochondrial fission, increase mtROS, reduce mitochondrial membrane potential, and alter mitophagy and mitochondrial respiration (111–113). Ozone exposure can increase mtROS and affect mitochondrial dynamics, mitophagy-related regulation, and respiratory-chain function (114, 115). Cigarette smoke can promote mitochondrial fragmentation and alter the expression of proteins involved in mitochondrial fission and fusion (116). Diesel exhaust particles can disrupt mitochondrial network integrity and reduce mitochondrial respiration (117). Toluene diisocyanate exposure can enhance fatty acid oxidation and increase mitochondrial oxidative stress (118). Inflammatory and immune signals, including TSLP, IL-4, IL-13, TNF-α, and IL-17, can alter mitochondrial structure, Ca^2+^ homeostasis, and mitophagy-related regulation (23, 58, 69, 91, 100). Metabolic abnormalities and other endogenous factors include obesity-associated disturbances in arginine–NO metabolism, 13(S)-hydroxyoctadecadienoic acid, and high mobility group box 1. Obesity-associated disruption of arginine–NO metabolism is associated with altered mitochondrial respiration and redox status, whereas 13(S)-hydroxyoctadecadienoic acid and high mobility group box 1 are linked to increased mitochondrial Ca^2+^ loading and altered endoplasmic reticulum–mitochondrial Ca^2+^ transfer, respectively (22, 51, 57).

Taken together, current evidence indicates that different asthma endotypes and phenotypes can exhibit distinct mitochondrial alterations, reflecting the complexity and heterogeneity of mitochondrial abnormalities in asthma. Different upstream stimuli can also induce distinct mitochondrial changes. Because direct comparisons among different asthma endotypes and phenotypes and among different upstream stimuli remain limited, further evidence is needed to better define these differences.

## 3 Mitochondria-derived damage signals and inflammatory sensing

Mitochondrial damage can generate or release several forms of mitochondria-derived signals that participate in inflammatory signaling. mtROS can regulate redox-sensitive signaling proteins, whereas mislocalized mitochondrial nucleic acids can engage corresponding innate immune sensing pathways. Oxidation, remodeling, and redistribution of mitochondrial membrane lipids, together with the release of mitochondrial proteins and other components, can also modify inflammation-related signaling. These mitochondria-derived damage-associated signals and signaling changes can thereby contribute to inflammatory gene transcription, nucleic acid sensing, and inflammasome activation, linking mitochondrial dysfunction to airway inflammation.

### 3.1 Mitochondrial reactive oxygen species signaling

Increased mtROS in asthma can modulate mitogen-activated protein kinase (MAPK)–activator protein 1 (AP-1), nuclear factor kappa B (NF-κB), and NLR family pyrin domain containing 3 (NLRP3) inflammasome signaling, thereby affecting inflammatory gene expression and inflammasome activation.

Different branches of MAPK signaling show distinct alterations in asthmatic airways. Comparisons among patients with mild asthma, patients with severe asthma, and healthy controls show that extracellular signal-regulated kinase 1/2 (ERK1/2) activation increases with disease severity and that p38 activation is enhanced in severe asthma, whereas c-Jun N-terminal kinase (JNK) phosphorylation is lower in both mild and severe asthma than in healthy controls (119). Phosphorylated p38 (p-p38) is increased in the bronchial submucosa of patients with mild asthma (120), and bronchial epithelial p-p38 levels are also higher in patients with more severe asthma than in healthy controls (121), further indicating abnormal p38 activation in asthmatic airways. Reducing mtROS can attenuate ERK1/2 and JNK–c-Jun/AP-1 activation. In primary human bronchial epithelial cells exposed to asthma-related stimuli, increased mtROS is accompanied by activation of ERK1/2, JNK, and c-Jun/AP-1. Reducing mtROS decreases AP-1 activation and the production of IL-6 and IL-8 (21). Treatment with the mitochondria-targeted superoxide scavenger MitoTEMPO reduces mtROS, accompanied by decreased ERK1/2, JNK, and AP-1 activation and reduced production of inflammatory mediators (122, 123). The association of mtROS with ERK1/2 and JNK–c-Jun/AP-1 is therefore supported by relatively direct intervention evidence, whereas p38, despite recurrent abnormal activation in asthmatic airways, currently lacks a comparable level of mtROS intervention evidence.

mtROS can modulate NF-κB activation. Mitochondria-targeted antioxidant intervention has shown that reducing mtROS can alter TNF-α-induced NF-κB activation, supporting a regulatory role for mtROS in this pathway (124). Persistent NF-κB activation is observed in a subset of patients with severe asthma. Bronchial epithelial transcriptomic analysis identified enrichment of NF-κB target genes in a subgroup of patients with poorer lung function, more frequent exacerbations, and higher corticosteroid requirements. Increased nuclear translocation of the NF-κB p65 subunit was also observed in airway epithelial brushings, and a similar transcriptional signature was reproduced in an independent cohort (125). Sputum transcriptomic profiles from neutrophilic and mixed-granulocytic severe asthma likewise show more prominent NF-κB signaling together with IL-1β- and TNF-α-related pathways (126). NF-κB activation also decreases when mtROS is reduced. Inhibition of mitochondrial Ca^2+^/calmodulin-dependent protein kinase II in airway epithelial cells reduces mtROS, accompanied by decreased p65 nuclear translocation and NF-κB transcriptional activity (127). Reducing respiratory chain complex II-associated ROS also decreases NF-κB activation and production of the chemokine CXCL14, while NF-κB inhibition similarly reduces CXCL14 secretion (128). Mechanistic evidence further shows that reducing mtROS attenuates phosphorylation of both ERK1/2 and p65 (46). These changes indicate that mtROS can influence NF-κB activation at the levels of p65 phosphorylation, nuclear translocation, and transcriptional activity.

mtROS can also promote NLRP3 inflammasome activation. In asthma-related airway inflammation, increased mtROS is accompanied by elevated NLRP3, caspase-1, and mature IL-1β, whereas inhibition of mtROS attenuates these changes (129). In fungal-associated airway inflammation, reduction of mtROS is likewise accompanied by attenuation of the NLRP3 inflammasome response (130). In HDM-induced airway inflammation, complex formation among NLRP3, apoptosis-associated speck-like protein containing a caspase activation and recruitment domain (ASC), and caspase-1, together with ASC oligomerization, has also been observed. Interventions that reduce mtROS concomitantly decrease inflammasome assembly (131). In human bronchial epithelial cells and cockroach allergen-associated asthma, direct reduction of mtROS likewise suppresses NLRP3 inflammasome activation (132). Further mechanistic evidence shows that mtROS can regulate IL-1β release during NLRP3 inflammasome activation (133). The association between mtROS and NLRP3 therefore extends beyond changes in NLRP3 abundance to inflammasome assembly, caspase-1 activation, and production of mature IL-1β.

mtROS can contribute to asthmatic airway inflammation by regulating inflammatory gene transcription and inflammasome activation. ERK1/2, JNK–c-Jun/AP-1, and NF-κB participate in the regulation of inflammatory gene expression, whereas NLRP3 links increased mtROS to inflammasome assembly, caspase-1 activation, and IL-1β maturation. p38 shows recurrent abnormal activation in asthmatic airways, but its direct association with mtROS is currently supported by less intervention evidence. The principal mtROS-responsive signaling pathways associated with inflammatory gene transcription and inflammasome activation in asthma are summarized in **Figure 7**.

**Figure 7:**
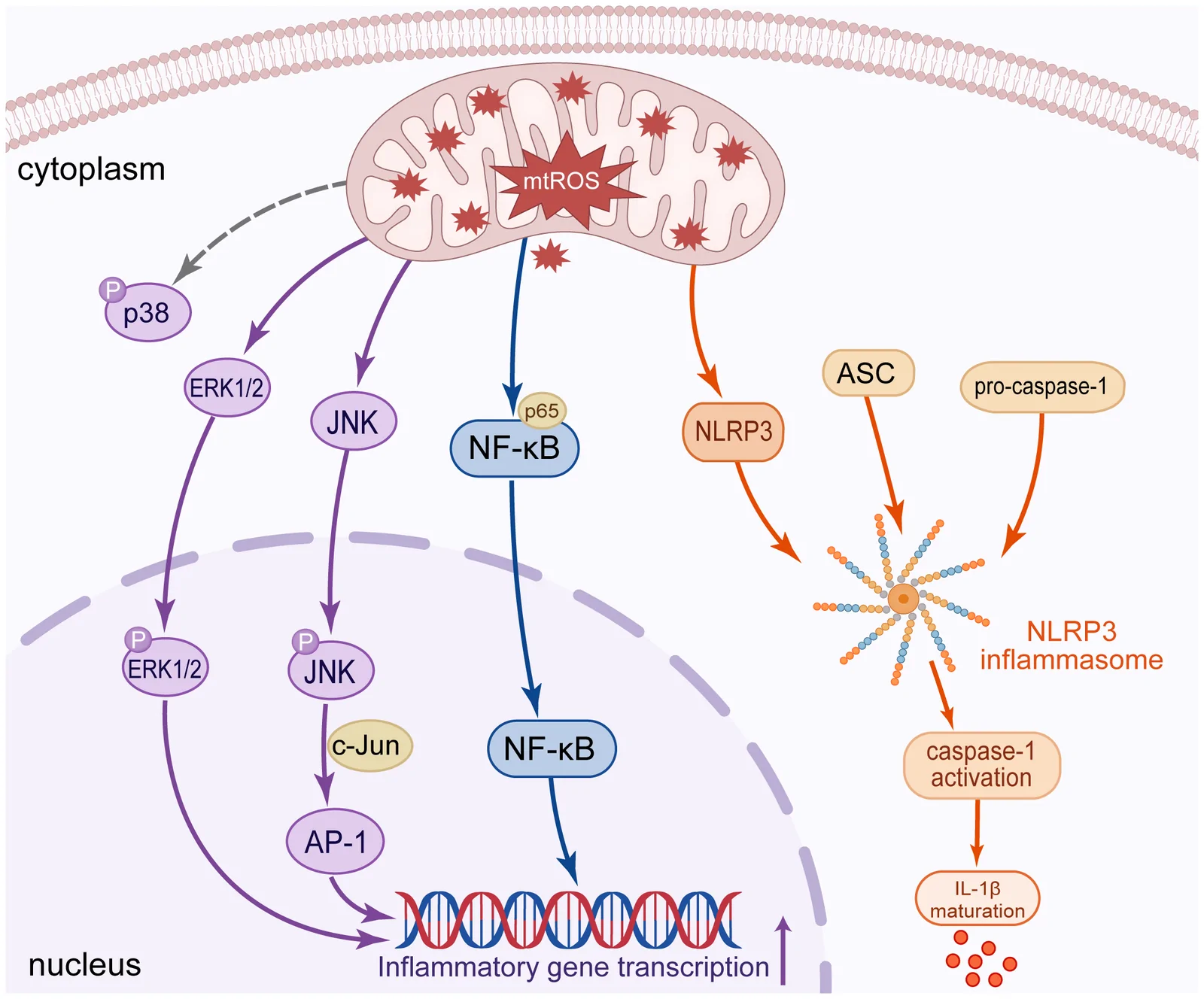
mtROS-responsive inflammatory signaling in asthma. Increased mtROS can modulate MAPK, NF-κB, and NLRP3 inflammasome signaling in asthma. mtROS-dependent regulation of ERK1/2 and JNK–c-Jun/AP-1 contributes to inflammatory gene transcription, whereas direct intervention evidence linking mtROS to p38 activation is more limited. mtROS also promotes NF-κB signaling, including p65 phosphorylation, nuclear translocation, and transcriptional activity. In parallel, increased mtROS promotes assembly of the NLRP3 inflammasome with ASC and pro-caspase-1, leading to caspase-1 activation and IL-1β maturation. AP-1, activator protein 1; ASC, apoptosis-associated speck-like protein containing a caspase activation and recruitment domain; ERK1/2, extracellular signal-regulated kinase 1/2; IL-1β, interleukin 1 beta; JNK, c-Jun N-terminal kinase; MAPK, mitogen-activated protein kinase; mtROS, mitochondrial reactive oxygen species; NF-κB, nuclear factor kappa B; NLRP3, NLR family pyrin domain containing 3; p65, NF-κB p65 subunit.

### 3.2 Mitochondrial nucleic acid signaling

Mitochondria contain mtDNA and mitochondrial RNAs transcribed from the mitochondrial genome. mtDNA associates with TFAM and other proteins to form nucleoids (134). Complementary transcripts generated by bidirectional transcription of mtDNA can form mitochondrial double-stranded RNA (mt-dsRNA), the abundance of which is regulated by mitochondrial RNA quality-control systems (135). Under physiological conditions, mitochondrial nucleic acids are confined within mitochondria and remain spatially separated from cytosolic nucleic acid sensors (136). When mitochondrial integrity and compartmentalization are disrupted, mtDNA can enter the cytosol or extracellular space, whereas mitochondrial RNA can gain access to the cytosol, allowing these nucleic acids to encounter sensing systems from which they are normally separated (137).

Cytosolic mtDNA can activate the cyclic GMP-AMP synthase (cGAS)–stimulator of interferon genes (STING) pathway. Upon sensing cytosolic DNA, cGAS generates cyclic GMP-AMP (cGAMP), which activates STING and downstream TANK-binding kinase 1 (TBK1)–interferon regulatory factor 3 (IRF3) and NF-κB signaling (138–140). Genetic deletion of cGAS attenuates experimental allergic airway inflammation (141). This pathway can also be enhanced when mitochondrial damage increases cytosolic mtDNA. In asthmatic airway epithelium, increased Parkin is associated with greater mtDNA release, whereas reducing Parkin decreases both mtDNA release and airway inflammation (142). Sustained opening of the mitochondrial permeability transition pore (mPTP) under HDM/lipopolysaccharide exposure likewise increases cytosolic mtDNA, whereas limiting mPTP opening reduces mtDNA release, cGAS–STING activation, and inflammatory responses (143). The asthma susceptibility gene *GSDMB* can further enhance mtDNA-induced cGAS–STING signaling in airway epithelial cells (144). In CD4^+^ T cells, loss of BLOC1S1 disrupts mitochondrial integrity, promotes mtDNA leakage, and activates cGAS–STING–NF-κB signaling, thereby favoring T helper 2 (Th2) polarization (145). These findings show that mtDNA released from damaged mitochondria can engage cGAS–STING-mediated DNA sensing. STING activation can subsequently influence asthma-related inflammatory responses. Exogenous cGAMP enhances type 2 allergic inflammation through STING–TBK1 signaling involving IRF3 and interferon regulatory factor 7, with subsequent IL-33-related signaling (146), whereas activation of STING by 2′3′-cGAMP can suppress IL-33-driven activation of group 2 innate lymphoid cells (ILC2s) and type 2 inflammation through IRF3–type I interferon signaling (147). Cytosolic DNA sensing by cGAS can therefore couple mtDNA escape to STING activation and subsequently influence type 2 airway inflammation.

Cytosolic mtDNA can also be sensed by absent in melanoma 2 (AIM2). In OVA-induced airway inflammation exacerbated by perfluorooctane sulfonate, mitochondrial damage is accompanied by cytosolic mtDNA accumulation, and mtDNA can be detected among AIM2-bound DNA. Reducing mtDNA abundance or loss of AIM2 attenuates inflammasome activation, and AIM2 deficiency also reduces airway inflammation (148). In addition to AIM2, cytosolic mtDNA can contribute to NLRP3 inflammasome activation. Oxidized mitochondrial DNA (ox-mtDNA) can directly bind to NLRP3 and promote inflammasome activation (149, 150). In asthma, mtDNA damage or release can occur together with NLRP3 inflammasome activation. In mice with neutrophilic asthma, reduced mtDNA integrity and content in lung tissue are accompanied by increased NLRP3, caspase-1, and IL-1β (129). In HDM-exposed human bronchial epithelial cells, mtDNA oxidative damage is accompanied by activation of the NLRP3–caspase-1–IL-1β pathway (74). In a subset of patients with acute asthma exacerbations, circulating mtDNA remains elevated together with a persistent IL-1β response; in human bronchial epithelial cells and peripheral blood monocytes from patients with asthma, NLRP3 inhibition reduces the release of both mtDNA and IL-1β (99). Thus, in addition to cGAS–STING-mediated DNA sensing, cytosolic mtDNA can activate the AIM2 inflammasome and contribute to NLRP3 inflammasome activation. However, direct binding of cytosolic ox-mtDNA to NLRP3 has not yet been demonstrated in asthma.

Unlike mtDNA, mt-dsRNA can be recognized by cytosolic RNA-sensing systems after entering the cytosol. Adenine nucleotide translocase 2 (ANT2) can mediate the transport of mitochondrial RNAs, including mt-dsRNA, across the inner mitochondrial membrane (151). Increased ETS transcription factor 2 (ETS2) has been observed in the airway epithelium of patients with asthma and in OVA-induced airway inflammation. ETS2 increases ANT2 expression and cytosolic mt-dsRNA and is accompanied by enhanced melanoma differentiation-associated protein 5 (MDA5)/retinoic acid-inducible gene I (RIG-I)-related responses. Reducing ETS2 or ANT2 decreases cytosolic mt-dsRNA and attenuates airway inflammation (152). Following cytosolic RNA recognition, MDA5 and RIG-I can transmit downstream signals through mitochondrial antiviral signaling protein (MAVS) on the outer mitochondrial membrane (153). Cytosolic mitochondrial RNA can therefore engage an RNA-sensing pathway distinct from mtDNA sensing.

After entering the extracellular space, mtDNA can also access endosomal nucleic acid sensing following cellular uptake. Pulmonary administration of mtDNA induces inflammation through toll-like receptor 9 (TLR9) in myeloid cells, whereas myeloid-specific TLR9 deficiency markedly attenuates pulmonary inflammation (154). In HDM-induced asthma, TLR9 deficiency or inhibition reduces airway hyperresponsiveness, eosinophilic inflammation, and type 2 cytokine production (155). Extracellular mtDNA can therefore engage TLR9-related endosomal sensing, while TLR9 also contributes to asthmatic airway inflammation.

Once displaced from their normal mitochondrial compartment, mitochondrial nucleic acids can engage different innate immune sensing pathways according to nucleic acid species, oxidation state, and location. Cytosolic mtDNA can enter cGAS–STING- and AIM2-related DNA-sensing pathways, and can also contribute to NLRP3 inflammasome activation, although direct binding of cytosolic ox-mtDNA to NLRP3 has not yet been demonstrated in asthma; cytosolic mt-dsRNA can engage MDA5/RIG-I–MAVS-related RNA sensing, and extracellular mtDNA can access TLR9-related endosomal sensing after cellular uptake. Mitochondrial nucleic acid mislocalization can therefore engage multiple innate immune signaling pathways involved in asthmatic airway inflammation, although the level of direct evidence differs among these pathways. The principal innate immune sensing pathways engaged by mislocalized mtDNA and mt-dsRNA are summarized in **Figure 8**.

**Figure 8:**
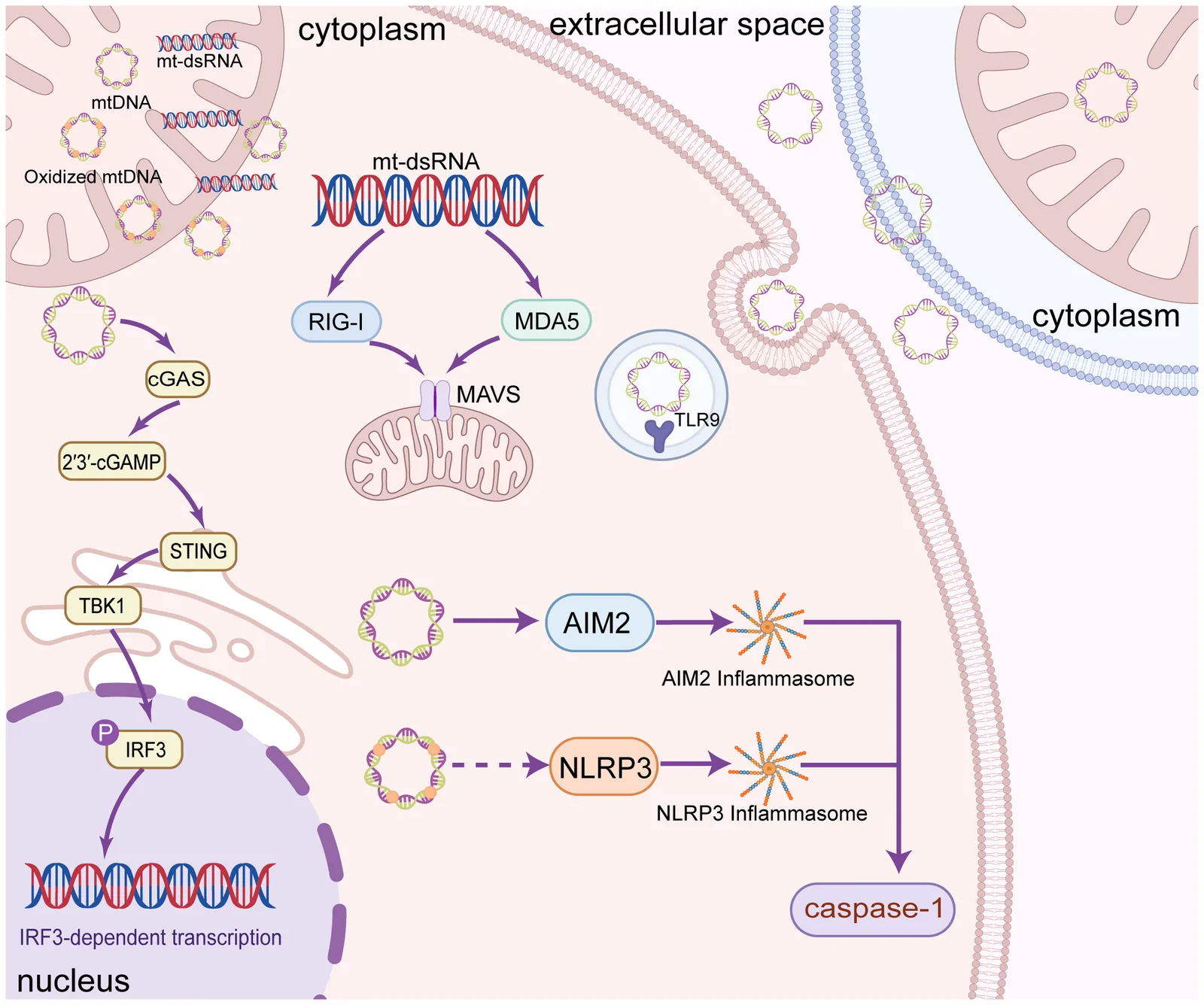
Innate immune sensing of mitochondrial nucleic acids in asthma. Mislocalized mitochondrial nucleic acids engage distinct innate immune sensing pathways according to nucleic acid species, oxidation state, and location. Cytosolic mtDNA activates cGAS, leading to 2′3′-cGAMP production and STING–TBK1–IRF3 signaling. Cytosolic mtDNA can also activate the AIM2 inflammasome, whereas oxidized mtDNA can promote NLRP3 inflammasome activation. Cytosolic mt-dsRNA is recognized by RIG-I and MDA5, which signal through mitochondrial MAVS. After release into the extracellular space and subsequent cellular uptake, mtDNA can access endosomal TLR9. These pathways link mitochondrial nucleic acid mislocalization to innate immune sensing in asthma. The dashed arrow indicates that direct binding of cytosolic ox-mtDNA to NLRP3 has been demonstrated in other inflammatory systems but has not yet been directly demonstrated in asthma. AIM2, absent in melanoma 2; cGAMP, cyclic GMP-AMP; cGAS, cyclic GMP-AMP synthase; IRF3, interferon regulatory factor 3; MAVS, mitochondrial antiviral signaling protein; MDA5, melanoma differentiation-associated protein 5; mtDNA, mitochondrial DNA; mt-dsRNA, mitochondrial double-stranded RNA; NLRP3, NLR family pyrin domain containing 3; RIG-I, retinoic acid-inducible gene I; STING, stimulator of interferon genes; TBK1, TANK-binding kinase 1; TLR9, toll-like receptor 9.

### 3.3 Mitochondrial membrane lipid and protein signaling

The inner and outer mitochondrial membranes have distinct lipid and protein compositions, and membrane phospholipids contribute to mitochondrial structure, respiratory protein activity, and protein transport (156). Oxidation, remodeling, or redistribution of membrane lipids can alter lipid–protein interactions, while mitochondrial damage can release proteins and other components from their normal compartments and expose them to cytosolic or extracellular signaling systems (157).

Mitochondrial membrane lipid alterations in asthma mainly involve PE oxidation and cardiolipin remodeling. Mitochondrial PE deficiency can impair OXPHOS and disrupt mitochondrial morphology (158). In asthmatic airway epithelium, 15LO1-dependent PE oxidation is increased and accompanied by mitochondrial injury (33). 15LO1-derived 15-HETE-PE promotes dissociation of RAF proto-oncogene serine/threonine-protein kinase (Raf-1) from PEBP1. Increased 15LO1 is also associated with reduced PEBP1–Raf-1 binding and enhanced ERK1/2 activation, whereas reducing 15LO1 restores PEBP1–Raf-1 binding and decreases ERK1/2 signaling and the expression of inflammation- and mucus-related genes (159). PEBP1 also binds 15LO1 and changes its substrate preference toward membrane-esterified PE, promoting the formation of hydroperoxy-PE species with pro-ferroptotic activity (160). Oxidized alkenyl ether-linked PE species are increased in airway epithelial cells from patients with severe asthma. In IL-13-treated human airway epithelial cells, these species accumulate mainly in the cytosolic rather than mitochondrial fraction, and their formation depends on 15LO1 but shows much less dependence on PEBP1 than diacyl oxidized PE species (161). PE oxidation in asthma therefore involves changes in PEBP1–Raf-1–ERK1/2 signaling together with the formation of oxidized PE species that differ in their dependence on PEBP1.

Altered cardiolipin remodeling has been observed in asthma. Fresh airway epithelial cells from patients with mild-to-moderate or severe asthma show increased levels of monolysocardiolipin, whereas oxidized cardiolipin remains low and is not significantly increased compared with healthy controls, showing accumulation of a remodeling intermediate without a corresponding increase in oxidized cardiolipin (33). Multi-omics analysis of lung tissue from HDM-sensitized mice also indicates dysregulation of cardiolipin-related pathways (162). Cardiolipin is a mitochondria-specific phospholipid located predominantly in the inner mitochondrial membrane (163). During mitochondrial damage, cardiolipin can translocate from the inner mitochondrial membrane to the surface of the outer mitochondrial membrane (164). Externalized cardiolipin can bind LC3 (165). Cardiolipin can also bind NLRP3 and promote NLRP3 inflammasome activation (166). In asthma, however, current evidence has mainly identified alterations in cardiolipin composition and remodeling, whereas cardiolipin translocation to the outer mitochondrial membrane and its binding to LC3 or NLRP3 have not yet been demonstrated.

Changes in heat shock protein 60 (HSP60) have also been reported in asthma. In alveolar macrophages from patients with allergic asthma, HSP60 is increased at the protein level despite reduced transcript levels, while the role of this intracellular accumulation remains unclear (167). In severe asthma, airway epithelial cells release HSP60. HSP60 exposure increases CCAAT/enhancer-binding protein beta (C/EBPβ) and ERK1/2 signaling in fibroblasts, followed by increased PRMT1 and PGC-1α expression. Bronchial thermoplasty reduces epithelial HSP60 release together with these signaling changes (168).

Mitochondrial membrane permeabilization can release cytochrome c (Cyt c) from its normal mitochondrial compartment. In toluene diisocyanate-sensitized and challenged asthmatic mice, phosphoglycerate mutase family member 5 (PGAM5) is increased in the airway epithelium. PGAM5 promotes BCL2-associated X protein (Bax) dephosphorylation at Ser184 and Bax translocation to mitochondria, followed by increased mitochondrial membrane permeabilization and Cyt c release. In human bronchial epithelial cells, reducing PGAM5 improves mitochondrial function and decreases mtROS and Cyt c release (169). Cyt c translocation from mitochondria to the cytosol has also been observed in OVA-induced asthmatic mice (39).

In steroid-insensitive asthma, mPTP opening promotes the release of multiple mitochondrial damage-associated molecular patterns (mtDAMPs) and activates the receptor for advanced glycation end products (RAGE). Exposure of human bronchial epithelial cells to released mtDAMPs also increases RAGE-related signaling. RAGE activation further increases mitochondrial injury and mPTP opening, followed by additional release of mtDAMPs, whereas inhibition of either mPTP or RAGE reduces this reciprocal amplification (170). Several mitochondrial components are released simultaneously during this process, and the component primarily responsible for RAGE activation has not been identified. In severe asthma, histidine metabolic reprogramming has also been associated with mitochondrial injury and a mitochondrially encoded NADH dehydrogenase 6-derived N-formyl peptide–formyl peptide receptor 2 pathway related to necroptotic signaling (171). The principal mitochondrial membrane lipid- and protein-related signaling pathways in asthma are summarized in **Figure 9**.

**Figure 9:**
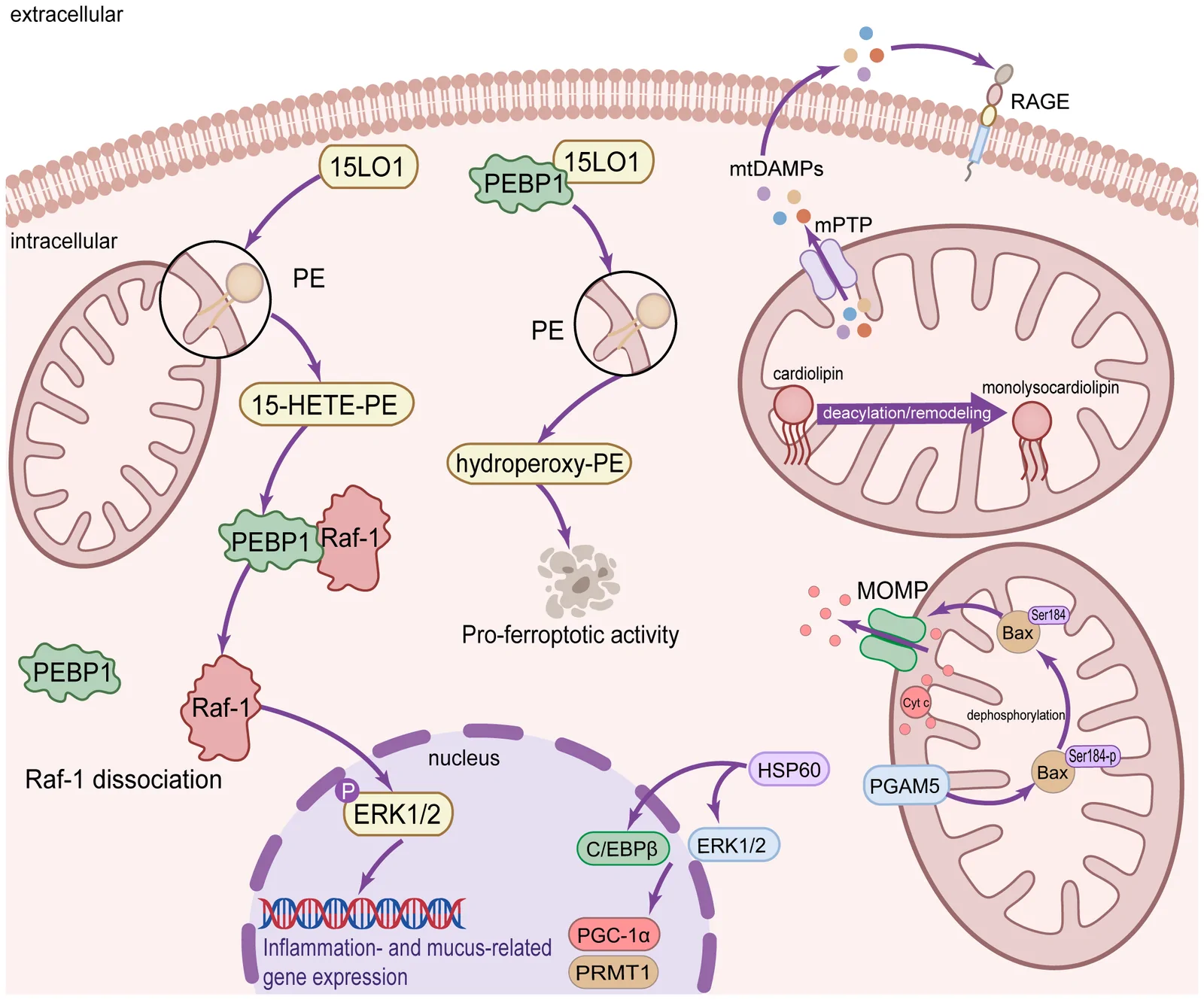
Mitochondrial membrane lipid and protein signaling in asthma. 15LO1-dependent oxidation of PE generates 15-HETE-PE, which promotes dissociation of Raf-1 from PEBP1 and is associated with ERK1/2 activation and the expression of inflammation- and mucus-related genes. PEBP1 also interacts with 15LO1 to promote the formation of hydroperoxy-PE species with pro-ferroptotic activity. Altered cardiolipin deacylation and remodeling in asthmatic airway epithelium are accompanied by increased monolysocardiolipin. HSP60 exposure increases C/EBPβ and ERK1/2 signaling, followed by increased PRMT1 and PGC-1α expression. PGAM5 promotes Bax dephosphorylation at Ser184 and mitochondrial translocation, leading to mitochondrial outer membrane permeabilization and Cyt c release. mPTP opening promotes the release of mtDAMPs and activation of RAGE-related signaling. 15-HETE-PE, 15-hydroxyeicosatetraenoic acid-phosphatidylethanolamine; 15LO1, 15-lipoxygenase-1; Bax, BCL2-associated X protein; C/EBPβ, CCAAT/enhancer-binding protein beta; Cyt c, cytochrome c; ERK1/2, extracellular signal-regulated kinase 1/2; HSP60, heat shock protein 60; MOMP, mitochondrial outer membrane permeabilization; mPTP, mitochondrial permeability transition pore; mtDAMPs, mitochondrial damage-associated molecular patterns; PE, phosphatidylethanolamine; PEBP1, phosphatidylethanolamine-binding protein 1; PGAM5, phosphoglycerate mutase family member 5; PGC-1α, peroxisome proliferator-activated receptor gamma coactivator 1-alpha; PRMT1, protein arginine methyltransferase 1; Raf-1, RAF proto-oncogene serine/threonine-protein kinase; RAGE, receptor for advanced glycation end products.

## 4 Impact of mitochondrial dysfunction on cell function in asthma

Mitochondrial abnormalities produce different functional consequences in immune cells and airway structural cells in asthma. In immune cells, these changes can alter cell generation and expansion, inflammatory mediator release, immune regulation, and survival. In airway structural cells, they can impair epithelial barrier integrity and repair, promote airway smooth muscle cell accumulation, and increase extracellular matrix production, thereby contributing to persistent airway inflammation and airway remodeling. The major cell-specific functional consequences of mitochondrial abnormalities in immune and airway structural cells are summarized in **Figure 10**.

**Figure 10:**
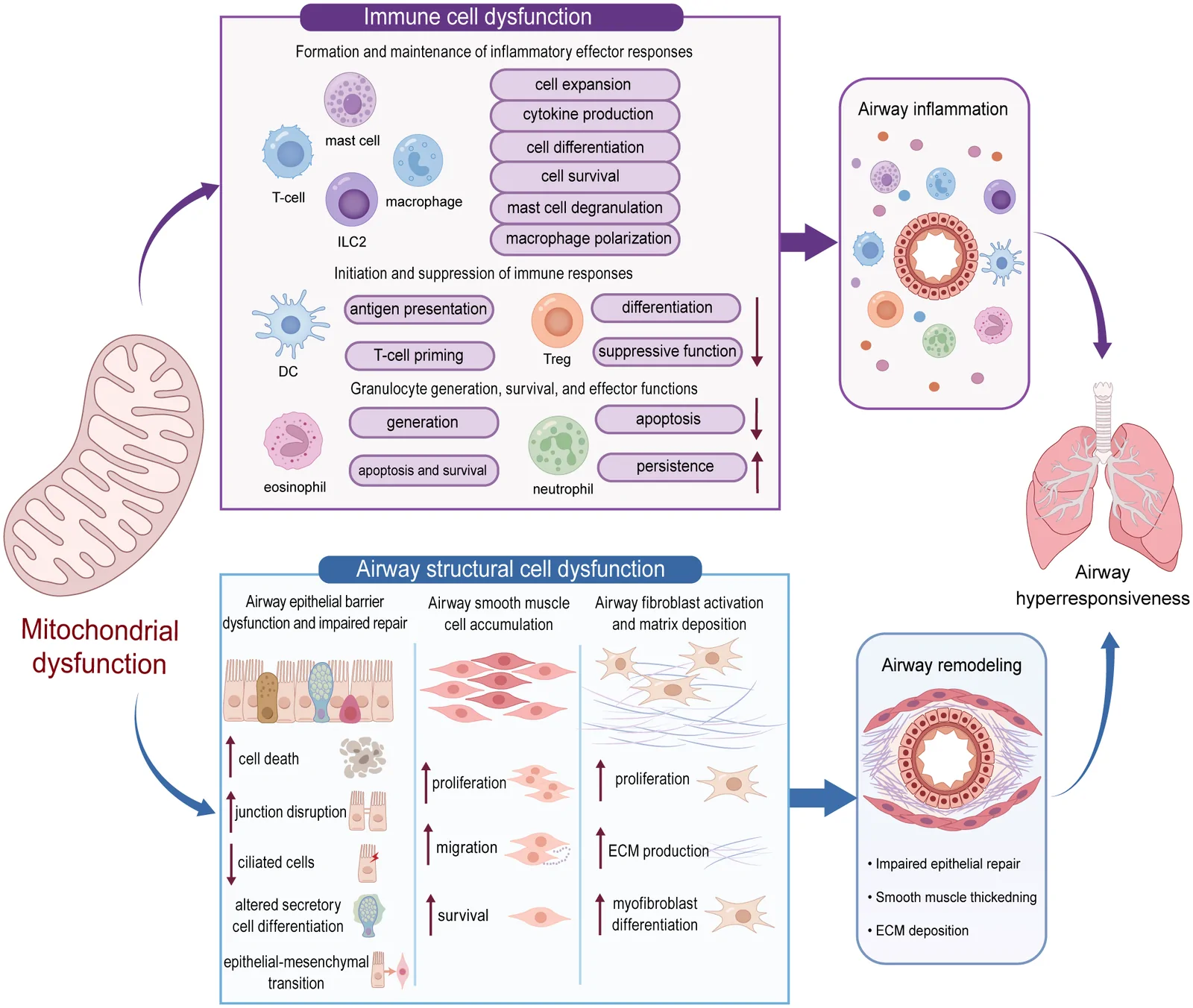
Cell-specific functional consequences of mitochondrial abnormalities in asthma. Mitochondrial abnormalities affect distinct functions across immune and airway structural cell populations. In ILC2s and effector T cells, mitochondrial metabolic and homeostatic changes influence cell expansion, differentiation, cytokine production, and survival. In mast cells and macrophages, mitochondrial changes affect degranulation and polarization, respectively. In DCs, mitochondrial redox and metabolic changes can alter antigen presentation and T-cell priming, whereas mitochondrial abnormalities in Tregs can impair differentiation and suppressive function. Granulocyte mitochondrial changes influence cell generation, apoptosis and survival, and persistence. In airway epithelial cells, mitochondrial abnormalities promote cell loss and junctional disruption and alter ciliated and secretory cell differentiation and epithelial cell state. In airway smooth muscle cells, mitochondrial changes support cell proliferation, migration, and survival, while in fibroblasts they influence proliferation, myofibroblast differentiation, and extracellular matrix production. These cell-specific functional changes contribute to persistent airway inflammation and airway remodeling. DC, dendritic cell; ECM, extracellular matrix; ILC2, group 2 innate lymphoid cell; Treg, regulatory T cell.

### 4.1 Immune cells

The functional consequences of mitochondrial abnormalities differ among asthma-related immune cell populations. In ILC2s and effector T cells, mitochondrial changes mainly affect cell expansion, cytokine production, and survival. In mast cells and macrophages, they influence degranulation, polarization, and inflammatory mediator release. In dendritic cells (DCs) and regulatory T cells (Tregs), mitochondrial changes affect the initiation and suppression of immune responses, respectively, while in granulocytes they alter cell generation, apoptosis, and persistence within the airways.

#### 4.1.1 Formation and maintenance of inflammatory effector responses

ILC2 expansion, cytokine production, and survival are closely linked to mitochondrial energy metabolism and homeostasis. Reduced mitochondrial respiration and ATP production decrease ILC2 proliferation and IL-5 and IL-13 production (172), while suppression of mitochondrial respiratory activity likewise attenuates their inflammatory activity (173). Conversely, enhanced mitochondrial respiration and ATP production increase ILC2 expansion and cytokine production, and subsequent inhibition of respiration reverses these effects (174). Restricted amino acid uptake reduces mitochondrial metabolism together with ILC2 expansion and cytokine production (175), while limited iron availability also decreases IL-5 and IL-13 production and the subsequent eosinophilic response (176). Impaired mitochondrial quality control is accompanied by damaged mitochondrial accumulation, reduced FAO, and increased ILC2 apoptosis (177). These mitochondrial changes therefore affect ILC2-mediated type 2 inflammation through changes in cell expansion, cytokine production, and survival.

Mitochondrial metabolism also influences the generation and sustained activity of effector T cells. The mitochondria-localized T-cell regulatory micropeptide interacts with pyrroline-5-carboxylate reductase 1 (PYCR1) to enhance glycolysis, thereby promoting Th2 differentiation and allergic airway inflammation (178). In established Th2 cells, reduced mitochondrial metabolic capacity is accompanied by impaired cell growth, proliferation, and survival, together with reduced production of IL-4, IL-5, and IL-13 (179). Redox defense also contributes to the maintenance of ILC2 and Th2 responses. In allergic airway inflammation, cysteine-driven GSH synthesis limits lipid peroxidation and ferroptosis and supports cell expansion; disruption of this antioxidant defense reduces ILC2 accumulation in the airways and attenuates type 2 inflammation (180). The relationship between mitochondrial metabolism and effector activity varies among other T-cell subsets. T helper 17 (Th17) dependence on glutaminolysis and mitochondrial respiration differs by sex, with Th17-mediated airway inflammation in female mice showing greater dependence on glutaminolysis (181). In Piezo1-deficient T helper 9 (Th9) cells, enhanced OXPHOS is accompanied by reduced IL-9 production and Th9 differentiation, and inhibition of this increase restores Th9 generation (182). In effector memory CD8^+^ T cells, enhanced mitochondrial metabolism increases survival while reducing interferon-γ-related effector activity (183). Mitochondrial transfer can also modify T-cell responses. Small extracellular vesicles released by airway myeloid regulatory cells from patients with asthma transfer functional mitochondria to CD4^+^ T cells, increasing mitochondrial content and metabolic activity in recipient cells and promoting antigen-specific T-cell activation and the generation of Th2, Th17, and Th2/Th17-like cells (76).

In mast cells, mitochondrial alterations mainly affect degranulation and inflammatory mediator release. Reducing mitochondrial ATP production decreases degranulation and exocytosis (184). Following activation, mitochondria move toward sites of exocytosis, and restricting this redistribution reduces degranulation and the release of preformed inflammatory mediators (185). Interventions targeting mitochondrial energy metabolism and signaling also reduce exocytosis and histamine release following IgE and antigen stimulation and attenuate airway inflammation (186). Mitochondrial energy supply and intracellular positioning therefore contribute to the release of granule contents after mast cell activation.

In macrophages, mitochondrial alterations mainly affect polarization and the maintenance of inflammatory states. Increased mitochondrial FAO is accompanied by enhanced OXPHOS and ATP production, increased M2-like polarization, and aggravated airway inflammation (187). M2-like polarization also increases when mitochondrial succinate dehydrogenase/respiratory chain complex II is inhibited and mtROS rises (188). Translocation of CD147 to mitochondria likewise promotes M2-like polarization (189). Myeloid TFAM deficiency reduces mitochondrial mass and mitochondrial gene expression while increasing cytosolic mtDNA and is accompanied by senescence-associated changes in macrophages and aggravated asthma-like inflammation (190). M2-like polarization can therefore occur under different mitochondrial metabolic states, while TFAM-associated mitochondrial impairment can also be accompanied by senescence-associated changes in macrophages.

#### 4.1.2 Initiation and suppression of immune responses

Mitochondrial changes in DCs can affect airway immune tolerance and the initiation of T-cell responses. Mitochondrial H_2_O_2_ contributes to the maintenance of airway immune tolerance, whereas reducing this signal increases lung DC numbers and enhances Th2 responses, eosinophilic airway inflammation, and airway hyperresponsiveness (191). Disturbances in DC mitochondrial metabolism can shift these cells toward stronger immune activation. Impaired itaconate metabolism increases antigen uptake, presentation, and T-cell priming (192), whereas disrupted mitochondrial fatty acid metabolism enhances antigen-presenting and costimulatory capacity (193). Thus, mitochondrial redox and metabolic changes can influence whether DCs maintain immune tolerance or promote allergic T-cell responses.

Mitochondrial abnormalities can impair Treg generation and suppressive function. Reduced mitochondrial metabolic activity and ATP production are accompanied by decreased IL-10 production and weaker suppression of effector T cells, whereas restoration of metabolic support partially restores Treg function and airway tolerance (194). Lysophosphatidylglycerol 18:0 treatment reduces mitochondrial membrane potential and ATP production and increases mtROS, together with impaired Treg differentiation and suppressive function (195). FAO further supports Treg generation, with enhanced FAO promoting Treg differentiation and inhibition of FAO reducing Treg formation (196).

#### 4.1.3 Granulocyte generation, survival, and effector functions

Mitochondrial alterations in eosinophils mainly affect cell generation and apoptosis. Iron chelation suppresses mitochondrial metabolic adaptation associated with eosinophil differentiation and reduces mature eosinophil generation and eosinophilic airway inflammation (197). Eosinophils from patients with asthma also show delayed spontaneous apoptosis and prolonged survival (198), whereas oxidant-mediated mitochondrial injury promotes eosinophil apoptosis (199). Thus, mitochondrial metabolism can influence eosinophil generation, while mitochondrial apoptotic signaling contributes to the regulation of eosinophil survival. Eosinophil effector activity also involves extracellular DNA traps. Eosinophils are capable of releasing mtDNA to form extracellular traps (200). Eosinophil extracellular DNA traps are also observed in asthmatic airways, where the extracellular DNA released by tissue-infiltrating eosinophils has been shown to be of mitochondrial rather than nuclear origin (201). Their formation capacity is increased in severe eosinophilic asthma, and these structures can promote degranulation and epithelial injury (202).

Mitochondrial apoptotic signaling also contributes to neutrophil survival. Prolonged neutrophil survival and impaired clearance have been observed in severe asthma in both adults and children (203, 204). In neutrophils from patients with atopic asthma, reduced translocation of pro-apoptotic signals to mitochondria is accompanied by decreased release of mitochondrial pro-apoptotic factors and weaker subsequent caspase-3 activation, resulting in delayed apoptosis (205). Attenuated mitochondria-mediated intrinsic apoptotic signaling may therefore contribute to prolonged neutrophil persistence in asthmatic airways.

Neutrophils can also exert effector functions by forming neutrophil extracellular traps (NETs). Mitochondria can contribute to NET formation, as neutrophils can release mtDNA to form NETs (206), while mtROS can also contribute to NET formation (207). Neutrophils from patients with severe asthma show increased NET formation, and the resulting NETs can induce eosinophil granule protein release (208). NETs can also contribute to the initiation and amplification of type 2 immune responses. In mice with allergic asthma, NETs promote allergen uptake by dendritic cells and enhance type 2 airway inflammation (209). During rhinovirus-induced asthma exacerbations, extracellular DNA released through NETosis can further amplify type 2 inflammation (210). These findings indicate that neutrophils can contribute to type 2 immune responses in asthma through NET formation, although a direct mechanistic link between increased NET formation and mitochondrial abnormalities in asthmatic neutrophils remains to be established.

### 4.2 Airway structural cell dysfunction and remodeling

Mitochondrial abnormalities in airway structural cells can impair epithelial barrier restoration, promote airway smooth muscle cell accumulation, and increase extracellular matrix production. In the airway epithelium, the major consequences include increased cell death, disruption of intercellular junctions, and impaired restoration of mucociliary structure. In airway smooth muscle, mitochondrial alterations mainly promote cell proliferation, migration, and survival, whereas in fibroblasts they can increase the number and prolong the survival of cells engaged in extracellular matrix synthesis. These functional changes contribute to epithelial barrier dysfunction, thickening of the airway smooth muscle layer, and extracellular matrix deposition, respectively.

#### 4.2.1 Airway epithelial barrier dysfunction and impaired repair

Mitochondrial abnormalities in airway epithelial cells can impair barrier integrity by increasing cell death and disrupting intercellular junctions. Limiting excessive mitochondrial Ca^2+^ uptake reduces IL-13-induced epithelial apoptosis and preserves barrier function (58). Restoration of impaired mitochondrial biogenesis restores junctional protein expression and increases transepithelial electrical resistance (92), while restoration of fatty acid utilization in epithelial cells from patients with severe asthma strengthens intercellular junctions and reduces permeability (46). Epithelial cell loss can also involve ferroptosis, which is attenuated by restoring 2,4-dienoyl-CoA reductase 1 expression (211). These findings link mitochondrial abnormalities to both epithelial cell loss and failure to maintain an intact intercellular barrier.

Mitochondrial changes also affect epithelial repair by altering ciliated and secretory cell differentiation and epithelial cell state. Mitochondria-associated ferroptotic injury reduces ciliated cell abundance and disrupts cilia development, whereas inhibition of this process improves the ciliary phenotype (33). Mitochondrial metabolism also influences secretory cell differentiation. Restricting mitochondrial pyruvate utilization in club cells reduces goblet cell differentiation (212), whereas loss of ARG2 increases goblet cell metaplasia and mucus production (47). Impaired intracellular mitochondrial transport further increases goblet cell numbers and intracellular mucus without a parallel increase in luminal mucin 5AC, indicating altered mucus handling as well as cell composition (81). PYCR1-dependent proline metabolism is also linked to epithelial–mesenchymal transition, and reducing proline synthesis or availability attenuates epithelial–mesenchymal transition and airway remodeling (213). Mitochondrial abnormalities can therefore impair epithelial repair through loss of ciliated cells, altered secretory cell differentiation, and changes in epithelial cell state.

#### 4.2.2 Airway smooth muscle cell expansion and remodeling

Mitochondrial alterations in asthmatic airway smooth muscle can increase smooth muscle cell accumulation within the airway wall by promoting cell proliferation and altering migration, adhesion, and apoptosis. Bronchial smooth muscle cells from patients with asthma show increased proliferation, which is reduced by depletion of mitochondrial content, while induction of mitochondrial biogenesis in cells from individuals without asthma also increases proliferation, further supporting a functional link between increased mitochondrial abundance and airway smooth muscle cell expansion (85). Asthma-derived airway smooth muscle cells also exhibit increased fatty acid uptake and utilization, and restricting this process markedly reduces cell proliferation (44). Increased mitochondrial abundance and fatty acid utilization can therefore support sustained expansion of airway smooth muscle cells in asthma.

Mitochondrial regulation also affects airway smooth muscle cell migration, adhesion, and apoptosis. Reducing BNIP3 decreases adhesion, migration, and proliferation of asthma-derived airway smooth muscle cells (214). Reducing NCLX expression decreases cell proliferation and migration while increasing apoptosis, and smooth muscle-specific NCLX deletion attenuates airway smooth muscle layer thickening and airway hyperresponsiveness (60). Current evidence therefore more directly links mitochondrial abnormalities to airway smooth muscle cell accumulation and smooth muscle layer thickening, whereas direct evidence that mitochondrial abnormalities increase the contractile capacity of individual airway smooth muscle cells remains limited.

#### 4.2.3 Airway fibroblast activation and matrix remodeling

Mitochondrial alterations in airway fibroblasts are associated with fibroblast proliferation, myofibroblast differentiation, and extracellular matrix production. Increased mitochondrial mass accompanies enhanced fibroblast proliferation and myofibroblast differentiation (89). Severe asthma-related epithelial signals similarly increase fibroblast proliferation and mitochondrial mass together with a marker of myofibroblast differentiation (168). These findings link increased mitochondrial abundance with expansion and differentiation of fibroblasts involved in extracellular matrix production.

Extracellular matrix production can also remain elevated when mitochondrial function declines. Bronchial fibroblasts from patients with severe asthma maintain high collagen expression despite reduced mitochondrial membrane potential and metabolic activity, without a marked increase in apoptosis or senescence (90). Further decline in mitochondrial function is accompanied by reduced apoptosis and increased collagen and fibronectin expression, while myofibroblast differentiation changes relatively little (91). Thus, persistent matrix production in severe asthma can coexist with reduced mitochondrial function and reduced apoptosis, even when myofibroblast differentiation shows little further change.

## 5 Therapeutic targeting of mitochondrial dysfunction in asthma

As understanding of the role of mitochondria in asthma has increased, mitochondria have also emerged as targets for therapeutic intervention. Current approaches targeting mitochondrial oxidative stress in asthma mainly include mitochondria-targeted antioxidants and redox-modulating compounds. MitoQ is a mitochondria-targeted antioxidant consisting of ubiquinone conjugated to a triphenylphosphonium cation, which enables its accumulation within mitochondria driven by the mitochondrial membrane potential and reduces mitochondrial oxidative damage (215). In mouse models of lean and obese allergic asthma, MitoQ attenuated airway hyperresponsiveness, inflammation, and remodeling, while the quinone-free mitochondria-targeting moiety dTPP^+^ produced some similar effects (216). In mice with steroid-insensitive asthma, MitoQ attenuated airway hyperresponsiveness, inflammation, and remodeling and improved airway epithelial mitochondrial function (217). A 12-week randomized, double-blind, placebo-controlled trial showed that MitoQ, compared with placebo, did not improve airway reactivity, asthma control, quality of life, or lung function in adults with obesity and poorly controlled asthma (218). Clinical evidence for MitoQ in asthma therefore remains limited, and its therapeutic effects require further evaluation in different patient populations. CoQ10, a component of the mitochondrial electron transport chain and an endogenous antioxidant, has also been used as adjunctive supplementation in patients with asthma. Available studies suggest that it may improve redox status and some measures of lung function, while combined antioxidant supplementation containing CoQ10 was associated with reduced corticosteroid requirements; however, the independent therapeutic effect of CoQ10 remains unclear (219, 220). AP39 is a mitochondria-targeted, slow-releasing hydrogen sulfide donor that reduces mitochondrial DNA damage and bioenergetic impairment in oxidatively stressed endothelial cells (221). In mice with allergic asthma, AP39 attenuated airway hyperresponsiveness and pulmonary inflammation and reduced eosinophil and lymphocyte levels as well as Th2 cytokine levels (222). Evidence for AP39 as a treatment for asthma therefore remains limited to preclinical studies.

Mitochondrial interventions in asthma can also act by protecting mitochondrial membrane structure and maintaining membrane homeostasis. SS-31 is a tetrapeptide that targets the mitochondrial inner membrane, binds cardiolipin, modulates the interaction between cytochrome c and cardiolipin, and maintains electron transport and ATP synthesis (223). In mice with steroid-insensitive asthma, SS-31 attenuated airway inflammation and mitochondrial dysfunction (224). In mice with chronic asthma, SS-31 reduced airway hyperresponsiveness and inflammation, decreased mucus secretion and subepithelial fibrosis, and suppressed cuproptosis-related changes in bronchial epithelial cells (225). However, evidence for SS-31 in asthma remains limited to preclinical studies, and its therapeutic efficacy and clinical applicability require further evaluation.

Improving mitochondrial substrate utilization and cellular energy metabolism may also be used as a therapeutic approach in asthma. L-carnitine is a cofactor required for the transport of long-chain fatty acids into mitochondria for β-oxidation and promotes fatty acid oxidation and energy production (226). A randomized, placebo-controlled study showed that L-carnitine supplementation improved asthma control and lung function in children with moderate persistent asthma (227). In asthmatic mice and airway epithelial cells, L-carnitine improved fatty acid oxidation and mitochondrial function and attenuated airway inflammation and epithelial barrier injury (228). Carnitine palmitoyltransferase 1A expression is reduced in the airway epithelium of patients with severe asthma, and L-carnitine restores fatty acid oxidation and mitochondrial energy metabolism and improves epithelial barrier function (46). Nicotinamide mononucleotide, a precursor of NAD^+^, attenuates airway inflammation, mucus secretion, and epithelial barrier injury by maintaining the stability of the mitochondrial deacetylase sirtuin 3, although the available evidence remains limited to preclinical studies (229). These findings suggest that metabolic modulation has therapeutic potential, although different interventions act on distinct metabolic processes, and their clinical evidence and appropriate patient populations in asthma remain to be defined.

Mitochondria-targeted delivery and nanotherapeutic approaches can improve drug delivery to mitochondria and enhance therapeutic effects in asthma. Nf-ABT-199 is a pH-sensitive nanoformulation carrying the selective BCL-2 inhibitor ABT-199. It enhances mitochondrial accumulation of the drug, promotes eosinophil apoptosis, and reduces airway inflammation and mucus secretion (230). HSA-TPP-PEG-PP10 is an inhalable mitochondria-targeted albumin nanoformulation loaded with PP10 that improves drug penetration through airway mucus and mitochondrial delivery and attenuates airway inflammation and mucus secretion in asthmatic mice (231). However, these mitochondria-targeted nanotherapeutic approaches remain limited to preclinical studies, and clinical studies in patients with asthma are still lacking.

Overall, therapeutic strategies targeting mitochondrial abnormalities in asthma remain at a relatively early stage. Only a small number of interventions have entered human studies. MitoQ has undergone a randomized controlled trial but has not demonstrated clear clinical benefit, whereas L-carnitine has shown improvements in asthma control or lung function in selected patient populations. Evidence for most mitochondria-targeted agents, metabolic modulators, and targeted delivery strategies still comes primarily from animal and cell studies. In addition, some compounds that modulate mitochondrial oxidative stress, membrane permeability, or other mitochondrial processes continue to be used mainly for mechanistic studies and lack clinical evidence as treatments for asthma. Current findings indicate that mitochondrial abnormalities in asthma are amenable to pharmacological intervention, although individual treatments act on different mitochondrial processes and their clinical effects may vary according to asthma phenotype and the specific mitochondrial abnormalities involved. Further studies are needed to identify the patients most likely to benefit from different mitochondrial interventions and to translate existing preclinical findings into human studies.

## 6 Discussion

Mitochondrial dysfunction in asthma is better understood as a disturbance of mitochondrial homeostasis rather than a uniform decline in mitochondrial function. Redox regulation, energy metabolism, Ca^2+^ homeostasis, mitochondrial dynamics, biogenesis, and quality control are closely interconnected, and alteration of one process can influence several others. Importantly, asthma-related studies have reported mitochondrial changes in different directions across metabolic, biogenesis, and quality-control processes. These findings argue against interpreting mitochondrial dysfunction as a uniform increase or decrease in mitochondrial activity. The key issue is therefore not whether mitochondrial activity is globally increased or decreased, but whether mitochondrial homeostasis remains sufficient to support normal cellular function while limiting the accumulation of damaged mitochondria.

The consequences of mitochondrial abnormalities depend to a large extent on the function of the affected cell. Inflammatory effector cells rely on mitochondria to support expansion, cytokine production, degranulation, and survival, whereas regulatory immune cells likewise depend on mitochondrial function to maintain suppressive activity. In airway structural cells, mitochondrial abnormalities more commonly affect epithelial repair, airway smooth muscle cell accumulation, and extracellular matrix production. The same mitochondrial process can therefore support inflammatory activity in one cell population while being required to maintain protective or regulatory function in another. These cell-specific differences help explain why apparently opposing findings can coexist across asthma studies. The pathogenic significance of a mitochondrial alteration should therefore be interpreted in relation to the cellular function it affects rather than from the direction of the mitochondrial change alone.

Persistent mitochondrial injury can also alter the role of mitochondria in inflammatory responses. Damaged mitochondria not only become less able to maintain cellular homeostasis but can also serve as sources of inflammatory signals. mtROS, displaced mitochondrial nucleic acids, oxidized or remodeled membrane lipids, and released mitochondrial proteins can participate in inflammatory gene transcription, nucleic acid sensing, and inflammasome activation. Mitochondrial injury can therefore extend from organelle dysfunction to signaling that influences inflammatory pathways and cellular behavior. At the same time, inflammatory mediators and oxidative stress can further disturb mitochondrial homeostasis. Mitochondrial dysfunction and airway inflammation may therefore reinforce one another, while mitochondrial abnormalities are also associated with impaired epithelial repair, inflammatory-cell persistence, airway smooth muscle cell accumulation, and extracellular matrix deposition.

The heterogeneity of mitochondrial abnormalities also has implications for the clinical translation of mitochondria-targeted interventions. Different asthma endotypes and phenotypes exhibit distinct mitochondrial alterations, while the interventions investigated to date act on different mitochondrial processes. Most mitochondria-related interventions remain supported primarily by preclinical studies, and human studies are still limited and have yielded variable results. Future studies should further define the predominant mitochondrial abnormalities in different patient populations and determine whether these features can help identify patients who may benefit from specific mitochondrial interventions, as well as whether such interventions produce clinically meaningful benefits.

What remains less well established is the continuity of this process within the same disease context. Current studies have separately linked mitochondrial injury to damage-signal generation, inflammatory sensing, and altered cellular function, but these relationships are usually supported by different cell types, disease phenotypes, and experimental systems. The temporal relationships among these events therefore remain unclear. This distinction is particularly important because the inflammatory environment itself continues to modify mitochondrial homeostasis, making it difficult to distinguish mitochondrial changes that contribute early to disease development from those that arise after inflammation is established. Studies that follow mitochondrial homeostatic disruption, damage-signal generation, and defined cellular consequences within the same disease context should help determine which mitochondrial alterations directly contribute to persistent inflammation and airway remodeling and which arise secondarily within an already altered inflammatory environment.
